# Co-targeting BET bromodomain BRD4 and RAC1 suppresses growth, stemness and tumorigenesis by disrupting the c-MYC-G9a-FTH1axis and downregulating HDAC1 in molecular subtypes of breast cancer

**DOI:** 10.7150/ijbs.62236

**Published:** 2021-10-25

**Authors:** Amjad Ali, Jasmin Shafarin, Hema Unnikannan, Nour Al-Jabi, Rola Abu Jabal, Khuloud Bajbouj, Jibran Sualeh Muhammad, Mawieh Hamad

**Affiliations:** 1 Research Institute of Medical and Health Sciences, University of Sharjah, Sharjah, United Arab Emirates.; 2 Department of Basic Medical Sciences, College of Medicine, University of Sharjah, Sharjah, United Arab Emirates.; 3 Department of Medical Laboratory Sciences, College of Health Sciences, University of Sharjah, Sharjah, United Arab Emirates.

**Keywords:** Breast cancer, BET bromodomain, BRD4, RAC1, c-MYC, FTH1, G9a, HDAC1, JQ1, NSC23766

## Abstract

BET bromodomain BRD4 and RAC1 oncogenes are considered important therapeutic targets for cancer and play key roles in tumorigenesis, survival and metastasis. However, combined inhibition of BRD4-RAC1 signaling pathways in different molecular subtypes of breast cancer including luminal-A, HER-2 positive and triple-negative breast (TNBC) largely remains unknown. Here, we demonstrated a new co-targeting strategy by combined inhibition of BRD4-RAC1 oncogenic signaling in different molecular subtypes of breast cancer in a context-dependent manner. We show that combined treatment of JQ1 (inhibitor of BRD4) and NSC23766 (inhibitor of RAC1) suppresses cell growth, clonogenic potential, cell migration and mammary stem cells expansion and induces autophagy and cellular senescence in molecular subtypes of breast cancer cells. Mechanistically, JQ1/NSC23766 combined treatment disrupts MYC/G9a axis and subsequently enhances FTH1 to exert antitumor effects. Furthermore, combined treatment targets HDAC1/Ac-H3K9 axis, thus suggesting a role of this combination in histone modification and chromatin modeling. C-MYC depletion and co-treatment with vitamin-C sensitizes different molecular subtypes of breast cancer cells to JQ1/NSC23766 combination and further reduces cell growth, cell migration and mammosphere formation. Importantly, co-targeting RAC1-BRD4 suppresses breast tumor growth *in vivo* using xenograft mouse model. Clinically, RAC1 and BRD4 expression positively correlates in breast cancer patient's samples and show high expression patterns across different molecular subtypes of breast cancer. Both RAC1 and BRD4 proteins predict poor survival in breast cancer patients. Taken together, our results suggest that combined inhibition of BRD4-RAC1 pathways represents a novel and potential therapeutic approach in different molecular subtypes of breast cancer and highlights the importance of co-targeting RAC1-BRD4 signaling in breast tumorigenesis via disruption of C-MYC/G9a/FTH1 axis and down regulation of HDAC1.

## Introduction

Breast cancer (BRCA) is among the leading causes of cancer-related deaths worldwide. BRCA patients with secondary metastasis often experience recurrence and relapse 1-3. Genetic and epigenetic changes in heterogeneous BRCA disease often lead to increased metastatic potential and mortality within 5 years of initial diagnosis 1-3. Although multiple chemotherapeutic agents are currently available to treat BRCA, the complex and heterogeneous nature of the disease continues to limit the efficacy of available drugs 3. To face this challenge, a growing collection of molecular targets is being constantly interrogated to develop novel experimental drugs that could expand the pool of potent anti-cancer drugs. One of the major targets that continues to hold promise in BRCA and other cancers is the c-MYC oncogene, which is deregulated in up to 50% of all cancers 4. The MYC oncoproteins, c-MYC in particular, are often referred to as “super-transcription factors” as they regulate the transcription of a wide array of genes that control multiple metabolic pathways 5. For example, c-MYC was recently reported to increase the intracellular labile iron pool by repressing the expression of FTH1, which is part of the cellular iron storing protein ferritin, and by stimulating the expression of the iron regulatory protein-2 (IRP2), which enhances iron influx to cancer cells 6.

BET (bromodomain and extra terminal domain) containing proteins including BRD2, BRD3 and BRD4 are chromatin remodeling epigenetic proteins that target acetylation-dependent histone modifications and hence play important roles in cancer progression, invasion and metastasis 7,8. BRD4 amplification was reported to transcriptionally activate c-MYC 9,10, with the consequent promotion of cancer growth, invasion and metastasis 11,12. JQ1, a small molecule inhibitor that blocks BRD4 and c-MYC expression and promotes apoptosis, was recently reported to suppress cancer cell invasion and migration as well as tumor metastasis 13,14. JQ1 was recently reported to suppress several oncogenic pathways including those related to angiogenesis and metastasis in different BRCA subtypes 15. Amplification of RAC1 (Ras-related C3 botulinum toxin substrate 1) has been shown to promote cancer metastasis, invasion, migration, epithelial to mesenchymal transition (EMT), angiogenesis, cell cycle progression and drug resistance 16. Increased expression of RAC1 has been documented in several human cancers and has been correlated with poor prognosis and decreased survival in cancer patients 17. RAC1 overexpression was recently shown to associate with multi-drug resistance to neoadjuvant chemotherapy by activating aldolase A and ERK signaling and enhancing glycolysis 18. Inhibition of RAC1 was previously reported to suppress cancer cell growth, invasion and tumor genesis 19 and was hence identified as a promising therapeutic target in different types of cancers 20-22. NSC23766, a promising inhibitor of RAC1 and muscarinic acetylcholine receptor (M_2_ mAChR) was shown to inhibit cell growth, invasion and migration in colorectal cancer 23,24. Use of endosomal pH-responsive nanoparticles to deliver RAC1-targeting siRNA along with cisplatin was recently reported to reverse NAC-chemoresistance in breast cancer patient-derived xenografts 18. Experimental evidences suggest that RAC1 and BRD4 oncogenes are also involved in regulating cellular iron metabolism. Epigenetic repression of BRD4 by JQ1 was recently reported to induce ferroptosis by altering the expression of multiple ferroptosis-associated genes 25. Additionally, treatment of various pancreatic cancer cell lines with the iron chelator deferasirox (DFX) reduced RAC1 and Cdc42 activity and suppressed cell motility 26. In this context, FTH1 as an iron storing protein 27,28 can inhibit the expression of oncogenic microRNAs in prostate cancer 29, promote migration and epithelial-mesenchymal transition (EMT) in breast, lung and ovary cancer 30,31 and enhance the transcriptional activity of p53 32. Increased FTH1 expression is now recognized a favorable prognostic marker in triple negative breast cancer (TNBC) 33.

We identified a novel and potential therapeutic strategy by co-targeting BET bromodomain BRD4 and RAC1 signaling pathways in different molecular subtypes of breast cancer including luminal-A, TNBC and HER-2 positive breast cancer. We show that combined inhibition of BRD4-RAC1 signaling pathways suppressed the tumor growth in MDA-MB-231 xenograft mouse model. Functionally, combination therapy suppressed the oncogenic potential of breast cancer cells, mammosphere formation and cell migration. Moreover, this combination also induced cellular senescence and autophagy in different molecular subtypes of breast cancer in a context dependent manner. Mechanistically, this combination therapy disrupted c-MYC-G9a-FTH1axis and revealed a potential molecular mechanism that might play an important role in breast tumorigenesis suppression. Combination therapy also targeted HDAC1/Ac-H3-K9 axis and thus points towards the role of this combination in histone modification and chromatin remodeling. Depletion of c-MYC further suppressed the cell growth, CSCs expansion and cell migration to combined treatment in context dependent manner. Co-treatment with Vitamin C showed further sensitivity to combined treatment in different molecular subtypes of breast cancer. RAC1 and BRD4 showed high expression in different molecular subtypes of breast cancer and high expression of both proteins correlated with decrease survival of breast cancer patients. Taken together, our data showed a novel potential therapeutic strategy by combined inhibition of BRD4-RAC1 oncogenic signaling pathways which decrease tumorigenesis and development of breast cancer by targeting c-MYC/G9a/FTH1 axis and HDCA1/Ac-H3K9 axis in different molecular subtypes in a context-dependent manner.

## Materials and Methods

### Cell culture

Human breast cancer cell lines MCF7, MDA-MB-231, SKBR3 (ATCC, Manassas, VA, USA) and JIMT1 (Cat. No. C0006005, AddexBio, San Diego, CA, USA) were used through this study. MCF-7 cell are luminal-like breast cancer cells that express estrogen receptor alpha (ERα), MDA-MB-231, MDA-MB-468 and BT-549 are triple negative breast cancer (TNBC) cells that do not typically express ERα, SKBR3 is a HER-2 positive cell line that does not typically express ERα, JIMT1 is a HER-2 positive cell line that is resistant to trastusumab. Additional human cell lines including the breast cancer cell line BT549 and the control cell lines 3T3 (mouse embryonic fibroblasts) and AC16 (human cardiomyocyte cell line) were used as a negative non-transformed cell line. Cells were grown in DMEM supplemented with 10% FBS and 1x PEST (Penicillin and streptomycin antibiotics) and incubated at 37 °C and 5% CO2 under humidified conditions. JQ1 (Cat. No. A1910), NSC23766 (NSC for short) (Cat. No. A1952), SP2509 (Cat. No. B4894), BRD4770 (Cat. No. B4837) and Ferrostatin-1 (Fer-1) (Cat. No. A4371) were purchased from ApexBio, Houston, TX, USA.

### Western Blotting

MCF-7, MDA-MB-231, SKBR3 and JIMT-1 BRCA cell treated with the indicated drug/drug combination for 72 h were pelleted, washed twice with cold 1X PBS and lysed with RIPA lysis buffer containing (150 mM sodium chloride, 1.0% NP-40, 0.1% SDS, 50 mM Tris pH 8.0, 0.5% sodium deoxycholate supplemented with protease inhibitors cocktail). Cell lysates were kept on ice for 15 minutes and then centrifuged at 4 °C for 15 minutes. Supernatants were collected and pellets were discarded; protein concentration in lysates was quantified by the Bradford method (Cat. No. 500-0006, Bio-Rad, Hercules, CA, USA). Samples were boiled in 5X loading buffer at 95oC for 5 minutes and boiled samples were loaded and run on 10% SDA-PAGE gel. Gel was transferred to PVDF membrane (Cat. No. 1620112, Bio-Rad) for overnight transfer prior to washing with 1X TBST and blocking in 5% non-fat dry milk (milk was dissolved in 1X TBST) for 1 h at room temperature. The membrane was washed 3 times with 1X TBST and sequentially but separately incubated with the primary antibody at 4oC overnight. Primary antibodies used in this study included those against FTH1 (LS-B11085-200; LS Bio, Seattle, WA, USA) (1:1000), GPX4 (A1933; Abclonal, Woburn, MA, USA) (1:500), SLC7A11 (A15604; Abclonal) (1:500), G9a/EHMT2 (D5R4R; Cell Singling Technology, Danvers, MA, USA) (1:1000) , NRF2 (D1Z9C; Cell Singling Technology) (1:1000), LC3B (D11; Cell singling Technology) (1:1000), c-MYC (D84C12; Cell singling Technology) (1:1000), LDHA (ab84716, Abcam, Cambridge, UK) (1:1000), HK1 (ab233837) (1:1000), PKM (ab61201) (1:1000) and β-actin (A 5441; Sigma-Aldrich, St. Louis, MO, USA) (1:2000), HDAC1 (A0238, Abclonal) (1:1000), Acetyl-Histone H3-K9 (A7255; Abclonal) (1:1000), LSD1/KDM1 (A15794; Abclonal) (1:1000), NOX4 (A11274; Abclonal) (1:1000), GLUT4 (A7637; Abclonal) (1:1000), GAPDH (D16H11; Cell Singling Technology) (1:2000), Hexokinase I (C35C4; Cell Singling Technology,) PKM1/2 (C103A3; Cell Singling Technology) (1:1000), LDHA (C4B5; Cell Singling Technology) (1:1000), Brd4 antibody ab128874 (1:1000). Primary antibodies were removed; membranes were washed 3 times with 1X TBST and incubated with secondary IgG antibodies. Secondary anti-mouse (Cat. No. 7076; Cell Signaling Technology) was reacted with the membrane at 1:1000 dilutions for 1 h at room temperature and the secondary anti-rabbit antibody (ac97040; Abcam) was reacted with the membrane at 1:5000 dilution for 1 h at room temperature. Secondary antibody was diluted in 3% BSA (dissolved in 1X TBST). After removal of the secondary antibody, membrane was washed 3 times with 1X TBST. Chemiluminescence was detected using the ECL kit (Cat. No. 32106; Thermo-Scientific). Protein band quantification was carried out using the Bio-Rad Image Lab software (ChemiDoc.Touch Gel and Western Blot Imaging System; Bio-Rad, Hercules, CA, USA); β- actin and GAPDH were used as a normalization control.

### Cell proliferation assay

Cell proliferation assay was performed using MTT 3-(4,5-dimethylthiazol-2-yl)-2,5-diphenyltetrazolium bromide) reagent (Sigma Aldrich). MDA-MB-231, JIMT-1, SKBR3 and MCF-7 cells were seeded in 96 well plates for 24 h prior to treatment with the indicated drug/indicated dose for an additional 72 h. 20 ul of MTT reagent was added to each well in media and incubated for 3 h at 37 °C under 5% CO2 humidified conditions. Media containing MTT reagent was then removed and 100 uL of DMSO was added/well to dissolve formazan crystals. Microplates were kept on a shaker for 5 minutes at room temperature to dissolve the formazan crystals in DMSO. Color intensity was measured/well using a 96 well plate reader [Crocodile mini Elisa reader; BioTeck, Winooski, VT, USA] at 590 nm. Averaged optical density readings were plotted against the type of treatment.

### siRNA knockdown

MDA-MB-231, JIMT1, SKBR3 and MCF7 cells were seeded and allowed to grow to 50-60% confluency for 24 h prior to transfection with control siRNA and siRNA targeting c-MYC using Lipofectamine RNAi-MAX Reagent. siRNA used included MYC siRNA (VHS40785; Cat. No. 1299001), FTH1 siRNA (s225998, Cat. No. 4392420), EHMT2/G9a siRNA (s21468, Cat. No. 4392420), Silencer™ Select GAPDH Positive Control siRNA (Cat. No. 4390849), all purchased from Thermo Fischer Scientific, Waltham, MA, USA). All Transfections were performed in Opti-MEM medium; transfection mixture was removed after 24 h and fresh medium containing 10% FBS was added to cells for 48 h. siRNA-silencing efficiency for both genes was confirmed by western blotting at 72 h post-transfection.

### Crystal violet and clonogenic assays

Cell viability and clonogenic potential of MDA-MB-231, JIMT-1, SKBR3 and MCF-7 cells transfected with control siRNA and siRNA targeting c-MYC and/or treated with indicated drug/drug combination were assessed by crystal violet and clonogenic assays. Cells were cultured in DMEM supplemented with 10% FBS and 1x PEST at 37 °C and 5% CO2 humidified conditions. For clonogenic assay, cells were treated with indicated drug concentrations for 72 h and then further maintained in drug-free medium for 14-18 days. Media was removed and cells were washed with 1X PBS. Cells were fixed with colony fixation solution (Acetic acid/Methanol (1:7) vol/vol) for 15 minutes at room temperature and then washed again carefully with 1X PBS. Cells were stained with 0.5% crystal violet at room temperature. For viability assessment with crystal violet, the dye was removed after 20 minutes and cells were washed carefully 3 times with distilled water to remove excess dye. Colony growth as indicated by crystal violet staining intensity was visualized by the naked eye.

Crystal violet staining absorbance: After staining cells with 0.5% crystal violet as described in the crystal violet staining assay section, a mixture of acetic acid and 50% ethanol (1:1) solution was added to each well to dissolve crystal violet absorbed by cells. Each independent reading absorbance was performed in triplicates and absorbance was measured at 570 nm using a 96 well plate reader [Crocodile mini Elisa reader; BioTeck, Winooski, VT, USA].

### Wound healing assay

MDA-MB-231, JIMT-1, SKBR3 and MCF-7 cells were seeded at 80-90% confluency for 24 h prior to treatment. A wound was created in fully confluent cultures using 10 ul sterile pipette tip; suspended cells were washed with 1X PBS. The respective drug was added to cells growing in culture media supplemented with 3% FBS. Wound healing area was determined at 0 and 24 h; the recovered surface area was calculated using the ImageJ software (https://imagej.nih.gov/ij/download.html).

### Senescence assay

MDA-MB-231, JIMT-1, SKBR3 and MCF-7 cells were plated in 24 well plates for 24 h prior to treatment with indicated drug concentration. After 72 h, media was removed and cells were maintained in drug-free medium for a total of 7 days. Media was removed and cells were washed with 1X PBS. Cells were fixed with 1X fixation solution for 15 minutes and washed again with 1X PBS. The β-galactosidase staining solution was prepared and added to cells following the manufacture's protocol (Cell Signaling Technology; kit #: 9860). Plates were covered with parafilm and incubated at 37 °C overnight with CO2 supply shut off. Parameters for the detection of senescent cells were adjusted and images were captured using confocal microscopy (Nikon, Tokyo, Japan).

### Mammosphere formation assay

MDA-MB-231 (1000 cells/well), JIMT-1 (1500 cells/well), SKBR3 (1500 cells/well) and MCF-7 (2000 cells/well) were grown in ultra-low attachment 96 wells plate (Corning Dow, Midland, Michigan, USA) as mammosphere. Mammosphere media was prepared using DMEM/F12 (Gibco, Waltham, Massachusetts, USA) supplemented with 1X B27 (Gibco); 10 ng/ml bFGF (Invitrogen, Carlsbad, CA), 20 ng/ml EGF (Sigma-Aldrich) and 1x PEST (Penicillin and streptomycin antibiotics). MDA-MB-231, JIMT-1, SKBR3 and MCF-7 cells formed mammosphere and were maintained in the incubator at 37 °C having 5% CO2. Cells were treated with DMSO, NSC, JQ1 or a combination of both and Vitamin C (Sigma-Aldrich) for indicated concentrations for 7 days and then bright-field images were taken using confocal microscopy (Nikon). As a measure of cancer stemness, percentage mammosphere formation efficiency (%MFE) was calculated based on the number of mammosphere formations with diameter >50 μm in the respective well using the formula: the number of mammospheres per well / number of cells seeded per well × 100 34.

### Scanning Electron Microscopy

MCF-7 cells were treated with DMSO as a vehicle, 20 uM NSC 1uM JQ1 or 20 uM NSC + 1uM JQ1 for 72 h. Cell pellets were fixed in 2.5% glutaraldehyde (G5882-Sigma) in 0.1 M Cacodylate buffer (C4945-sigma) at pH 7.3, supplemented with 2% sucrose (84097-sigma) for 1 h. Cells were washed once with the same cacodylate buffer. Cells were further fixed with 1% Osmium tetroxide in cacodylate buffer for 30 min. Cells were then washed with cacodylate and dehydrated through graded ethanol concentrations. After processing, samples cells were dried using a critical point dryer. All cells were coated with gold (Sputter Coating System, Quorum Technology Mini Sputter Coater, SC7620) and examined by scanning electron microscopy (Tescan VEGA XM variable pressure SEM, Czech Republic).

### Xenograft nude mouse model studies

Animal studies were performed according to a protocol that was reviewed and approved by the Animal Care and Use Committee (ACUC), University of Sharjah. Nine adults (4-6 weeks of age) female BALB/c nude mice raised under specific-pathogen-free (SPF) conditions at the University of Sharjah vivarium were used. ~106 MDA-MB-231 cells mixed at 1:1 ratio with BD matrigel basement membrane matrix were transplanted into both flanks of mice. Mice bearing tumors >50 mm^3^ (9 in total) received 50 µl (i) DMSO (vehicle) or an equal volume of (ii) NSC at 30 mg/Kg, (iii) JQ1 at 20 mg/Kg, or (iv) a combination of both through the intraperitoneal route. Each mouse group received the respective treatment 3 times per week for 3 weeks. Tumor volume and mouse body weight were periodically measured (3 times per week for 3 weeks). Throughout the study period, the general health of the mice was closely monitored should signs of severe sickness necessitating merciful sacrifice manifest; no such action was needed. All mice were sacrificed at day 21 post the start of the experiment; mice were then photographed and tumors from both sides were removed for further analysis.

### Cancer bioinformatics analysis

Expression and Survival analysis of RAC1 and BRD4 were performed in BRCA tissue samples using PrognoScan database (http://dna00.bio.kyutech.ac.jp/PrognoScan/) 35, Breast Cancer Gene-Expression Miner v4.4 database (http://bcgenex.centregauducheau.fr/BC-GEM/GEM-Accueil.php?js=1) 36 and DriverDBv3 database (http://driverdb.tms.cmu.edu.tw/gene?symbol=PIP5K1A&tab=miRNA). Tumor cells infiltration and SCNA analysis for RAC1 and BRD4 were performed using TIMER database (https://cistrome.shinyapps.io/timer/) 37 Expression pattern analysis of RAC1 and BRD4 across different molecular subtypes of BRCA, histological types of BRCA and nodal status were performed using Breast Cancer Gene-Expression Miner v4.4 database.

### Statistical analysis

Student t test was used for statistical analysis of cell viability, cell survival, mammosphere assay, wound healing assay, senescence assay and tumor weight data. Student t-test statistics was used to analyze tumor volume and mouse weight data. Pearson's correlation coefficient was used for statistical analysis of data generated using the bc-Genex miner 4.0. Log-rank test was used to analyze data generated using the Cbioportal (R package from Cbioportal). Spearman's test was used for the correlation analysis of data generated using the TIMER database. P<0.05 was considered as statistically significant (*p<0.05, **p<0.005, ***p<0.0005 and #p>0.05); statistical analysis was performed using the GraphPad Prism 7 software.

## Results

### Co-targeting RAC1 and BRD4 reduces growth and clonogenic potential of different molecular subtypes of BRCA

**Figure 1:**
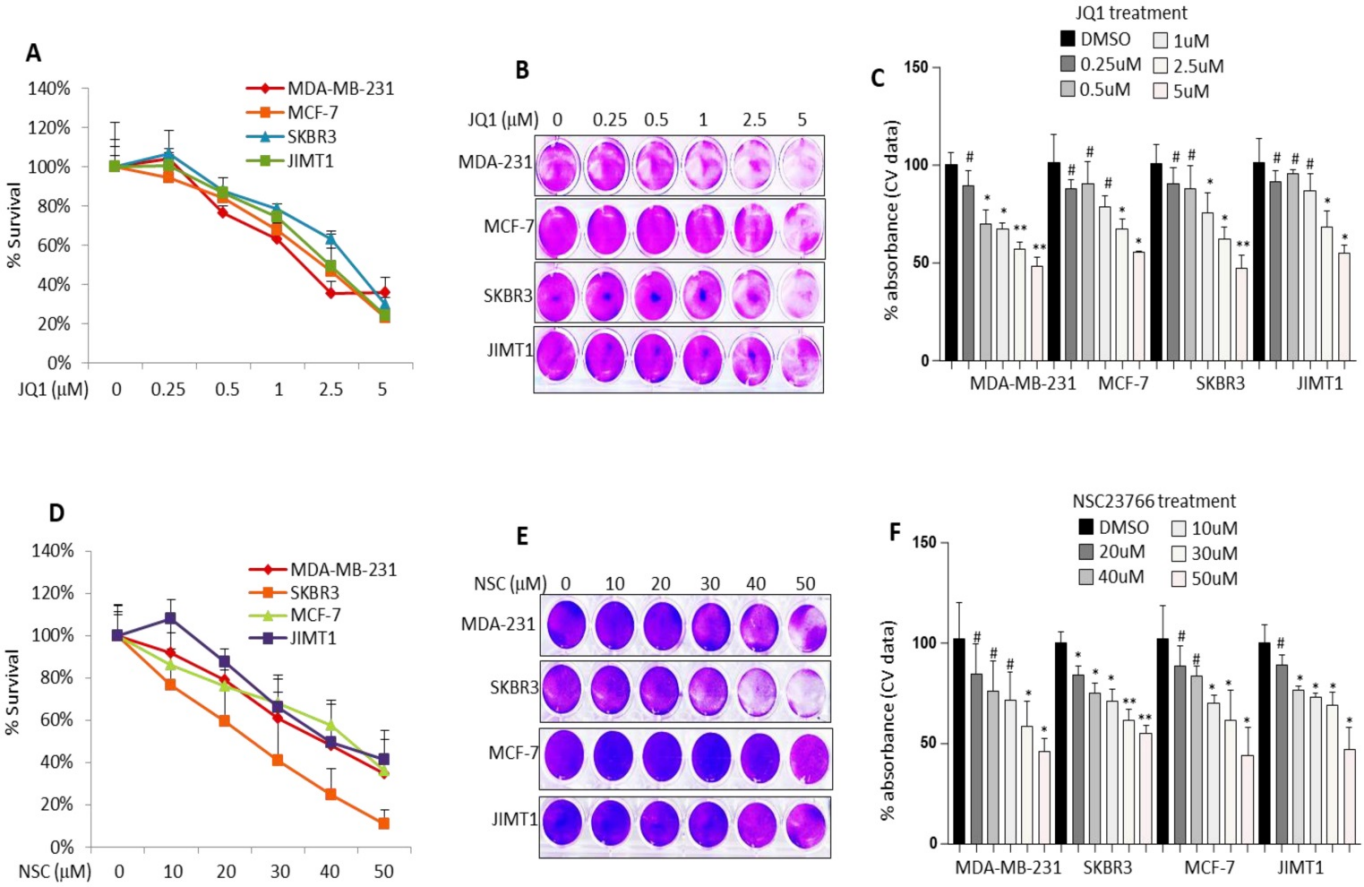
** Treatment with JQ1 or NSC inhibits different molecular subtypes of BRCA cell growth.** MCF-7, MDA-MB-231, SKBR3 and JIMT-1 cells were treated with increasing concentrations of JQ1 for 72 h and assessed for viability by (**A**) MTT assay and (**B**) crystal violet staining. **(C)** Mean ± SD of crystal violet absorbance values based on three independent experiments. MCF-7, MDA-MB-231, SKBR3 and JIMT-1 cells were treated with increasing concentrations of NSC for 72 h and assessed for viability by (**D**) MTT assay and (**E**) crystal violet staining. **(F)** Mean ± SD of crystal violet absorbance values based on three independent experiments. Data presented is the mean ± SD of three independent experiments; *p < 0.05; **p < 0.005; ***p < 0.0005; # no significant difference.

**Figure 2:**
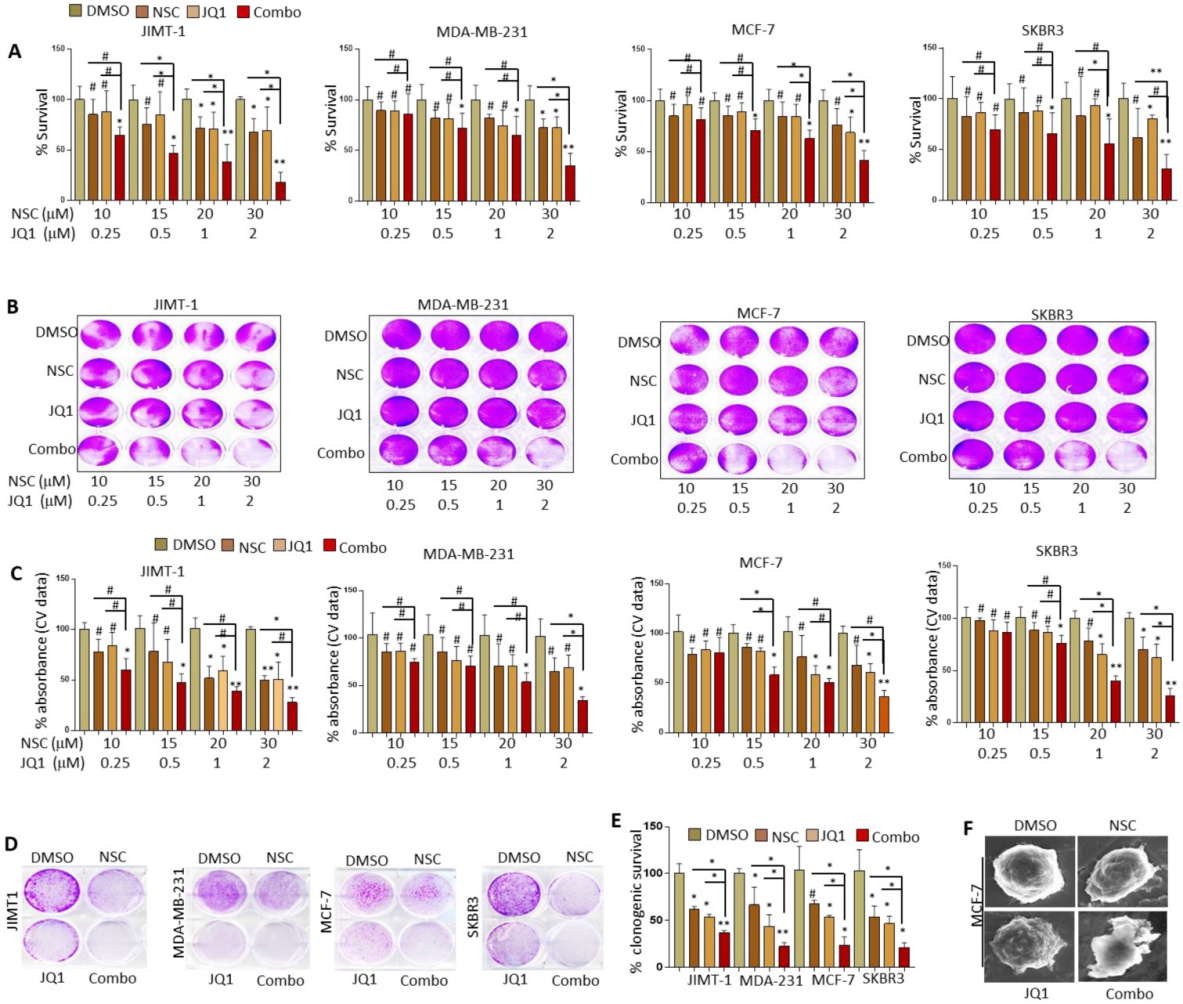
** Combined JQ1 plus NSC treatment suppresses growth and oncogenic potential of different molecular subtypes of BRCA cells.** MCF-7, MDA-MB-231, SKBR3 and JIMT-1 cells were treated with increasing concentrations of JQ1 and/or NSC for 72 h and assessed for viability by (**A**) MTT assay and (**B**) crystal violet staining. **(C)** Mean ± SD of crystal violet absorbance values based on three independent experiments. Data presented in A and C is the mean ±SD of three independent experiments; *p < 0.05; **p < 0.005; ***p < 0.0005; # not significant. **(D)** MCF-7, MDA-MB-231, SKBR3 and JIMT-1 cells were treated with JQ1 (0.5 uM) and/or NSC (15 uM) for 72 h and further grown in drug-free medium for 14-18 days; clonogenic potential was assessed using crystal violet staining. **(E)** Graph represents statistical analysis of clonogenic data. *p < 0.05; **p < 0.005; ***p < 0.0005; # not significant. **(F)** SEM micrographs of MCF-7 cells treated with DMSO, JQ1 (2 uM) and/or NSC (30 uM) for 72 h and photographs were taken.

Based on preliminary data pertaining to the clinicopathological significance of RAC1 and BRD4 expression in BRCA the effects of NSC (RAC1 inhibitor) and JQ1 (BRD4 inhibitor) on cell growth and migration was investigated in MCF-7, MDA-MB-231, JIMT1 and SKBR3 BRCA cell lines. Treatment with increasing concentrations of JQ1 or NSC resulted in a dose-dependent decrease in cell growth as assessed by MTT and crystal violet staining irrespective of cell type (Fig. 1A-F). Co-targeting RAC1 and BRD4 resulted in significantly lower levels of cell viability as compared with that in untreated controls or cells separately treated with NSC or JQ1, especially at [20 uM NSC + 1uM JQ1] and at [30 uM NSC + 2uM JQ1] (Fig. 2A). The combination index (CI) for MCF-7, MDA-MB-231, JIMT1 and SKBR3 cells treated with NSC and/or JQ1 was also calculated (Table 1). For the most part, the NSC+JQ1 drug combination resulted in synergistic effects especially in JIMT1 and SKBR3 and especially and more clearly at higher concentrations. Weak antagonistic effects of the drug combination were observed in MDA-MB-231 and MCF-7 cells especially at low drug concentrations. A similar pattern of growth inhibition was observed by crystal violet staining (Figs. 2B and 2C). Treatment of additional cell lines including the cancerous cell lines BT549 and MDA-MB-468 with NSC or JQ1 alone or in combination showed that cells treated with both inhibitors showed significantly reduced growth, especially at high doses (30 uM NSC + 2 uM JQ1) relative to those treated with either agent alone (Figs. S1A-S1E). Minimal growth inhibition was observed in the primary cell lines 3T3 and AC-16 with NSC and/or JQ1 following treatment with different doses of NSC and/or JQ1 (Figs. S2A-S2F). Treatment of MCF-7, MDA-MB-231, SKBR3 and JIMT-1 cells with JQ1 (0.5 uM) or NSC (15 uM) for 72 h followed by continued growth of treated cells in drug-free medium for 14-18 days resulted in a significant reduction in clonogenicity, especially in JIMT1 and SKBR3 cells (Fig. 2D). Moreover, the clonogenic potential was significantly reduced in MCF-7, MDA-MB-231, SKBR3 and JIMT-1 cells treated with “0.5 uM JQ1 + 15 uM NSC” relative to that in counterparts treated with either agent alone (Fig. 2E). Morphological evaluation of the effects of NSC (20 µM) and/or JQ1 (1 µM) treatment on cell membrane integrity in MCF-7 cells was further investigated by SEM. Interestingly, treatment with NSC and/or JQ1 resulted in significant membrane blebbing in MCF-7 cells (Fig. 2F). However, the degree of membrane damage resulting from combined (20 uM NSC and or 1 uM JQ1) treatment was more pronounced than in cells treated with either agent alone. Taken together, these results suggest that combined inhibition of both RAC1 and BRD4 suppresses breast cancer cell growth and clonogenic potential. These results also highlight the importance of co-targeting RAC1-BRD4 axis in breast cancer progression to reduces tumor burden.

### Co-targeting BRD4-RAC1 induces cell senescence, reduces CSC-like potential and suppresses cell migration in different molecular subtypes of BRCA

**Figure 3:**
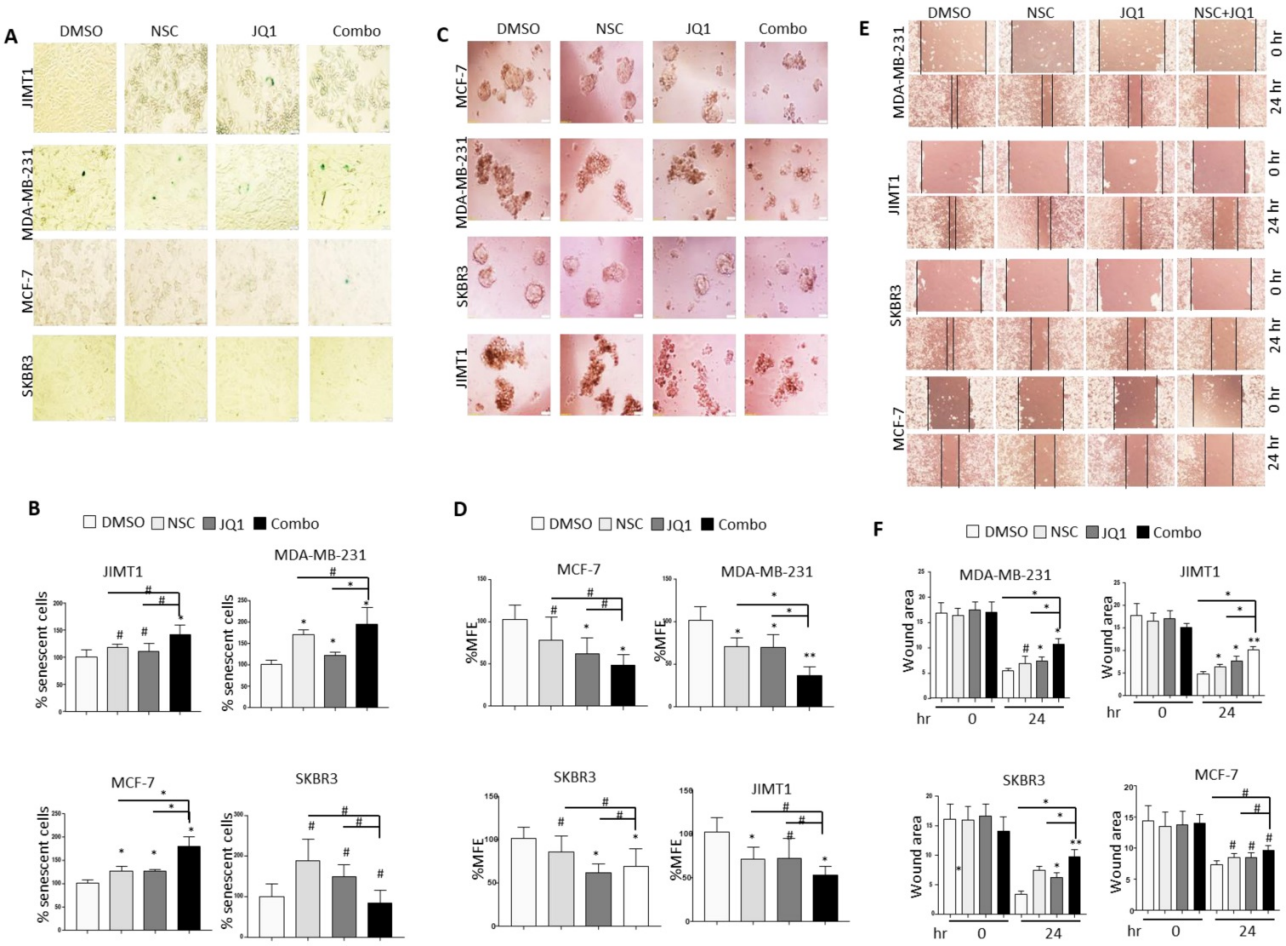
** Combined JQ1 plus NSC treatment induces cellular senescence, inhibits mammosphere formation and cell migration in breast cancer cells. (A)** MCF-7, MDA-MB-231, SKBR3 and JIMT-1 cells were treated with JQ1 (0.5 uM) and/or NSC (15 uM) for 72 h and further grown in drug-free medium for 7 days to determine cellular senescence. **(B)** Statistical analysis of data from A; data presented is the mean ±SD of three independent experiments. *p < 0.05; **p < 0.005; ***p < 0.0005; # not significant. **(C)** MCF-7, MDA-MB-231, SKBR3 and JIMT-1 cells were grown in ultra-low attachment plates and treated with JQ1 (0.5 uM) and/or NSC (15 uM) for 7 days to examine mammosphere formation. **(D)** Mean ±SD of mammosphere formation efficiency based on three independent experiments. *p < 0.05; **p < 0.005; ***p < 0.0005; # not significant. **(E)** MCF-7, MDA-MB-231, SKBR3 and JIMT-1 cells were treated with JQ1 (0.5 uM) and/or NSC (15 uM) for 72 h prior to performing the wound healing assay as means of determining cell migration potential. Wound healing area was measured at 24 h post scratching; images shown are representative of three independent experiments. **(F)** Histograms next to corresponding images is the mean ±SD of wound healing potential based on three independent experiments. *p < 0.05; **p < 0.005; ***p < 0.0005; # not significant.

To further investigate the anti-tumor effects of co-inhibiting BRD4 and RAC1, cells treated with JQ1 and/or NSC were evaluated for cellular senescence, migration and CSC-like phenotype. MCF-7, MDA-MB-231, JIMT1 and SKBR3 cells treated with JQ1 or NSC for 7 days showed significantly higher levels of senescence relative to controls. Additionally, MCF-7, MDA-MB-231 and JIMT1 cells treated with JQ1 and NSC for 7 days showed significantly higher levels of senescence relative to that in controls or cells treated with either agent alone (Fig. 3A-B). However, levels of cellular senescence in SKBR3 treated with JQ1 and NSC were significantly lower than that in counterparts treated with either agent alone. CSC-like features in different types of human cancer have been linked to tumor metastasis and reduced patient survival, perhaps due to resistance to chemotherapy and continuous tumor progression 38,39. To test whether co-targeting BRD4 and RAC1 reduces CSC-like potential in BRCA, cells seeded in ultra-low attachment plates and treated with JQ1 and/or NSC for 7 days were examined for mammosphere formation efficiency (MFE; a measure of stemness). As shown in (Fig. 3C-D), treatment with JQ1+NSC resulted in a significant reduction in MFE. Reduced MFE following treatment with JQ1+NSC was more pronounced in MCF-7 and MDA-MB-231 cultures as compared with that in SKBR3 and JIMT1. Of note here is that both SKBR3 and JIMT1 are HER2+ and that amplification of HER2 was reported to promote stemness or CSC-like features 38. It is well established that increased cancer cell migration potential contributes to invasion and metastasis; hence the potential therapeutic utility of agents that reduce cell migration potential in pre-metastatic/metastatic tumors 40. In this context, the possibility that co-targeting RAC1 and BRD4 might reduce the migration potential of BRCA cells was addressed using the wound healing assay. As shown in Fig. 3E and 3F, treatment of MDA-MB-231, JIMT1 and SKBR3 cells with JQ1 or NSC resulted in a significant reduction in cell migration potential relative to that in DMSO-treated cells. Moreover, treatment of MDA-MB-231, JIMT1 and SKBR3 cells with JQ1+NSC resulted in a significant decrease in cell migration relative to that in DMSO-treated cells or cells treated with either agent alone. Migration inhibition in JQ1+NSC-treated MCF-7 cells was not as significant as that in other cell lines. It is worth noting here that, unlike the rest of the cell lines used in this study, MCF-7 cells express high levels of ERα, which plays a significant role in cell migration. Therefore, it is tempting to speculate that ERα-mediated signaling may lie behind the differences we observed, vis-à-vis absence of a clear effect of JQ1+NSC treatment on MCF-7 cell migration. Taken together, these results suggest that combined inhibition of RAC1-BRD4 signaling promote cellular senescence, inhibits mammopshere formation and cell migration in different molecular subtypes of breast cancer.

### Co-targeting RAC1-BRD4 signaling suppresses breast tumor growth *in vivo*

**Figure 4:**
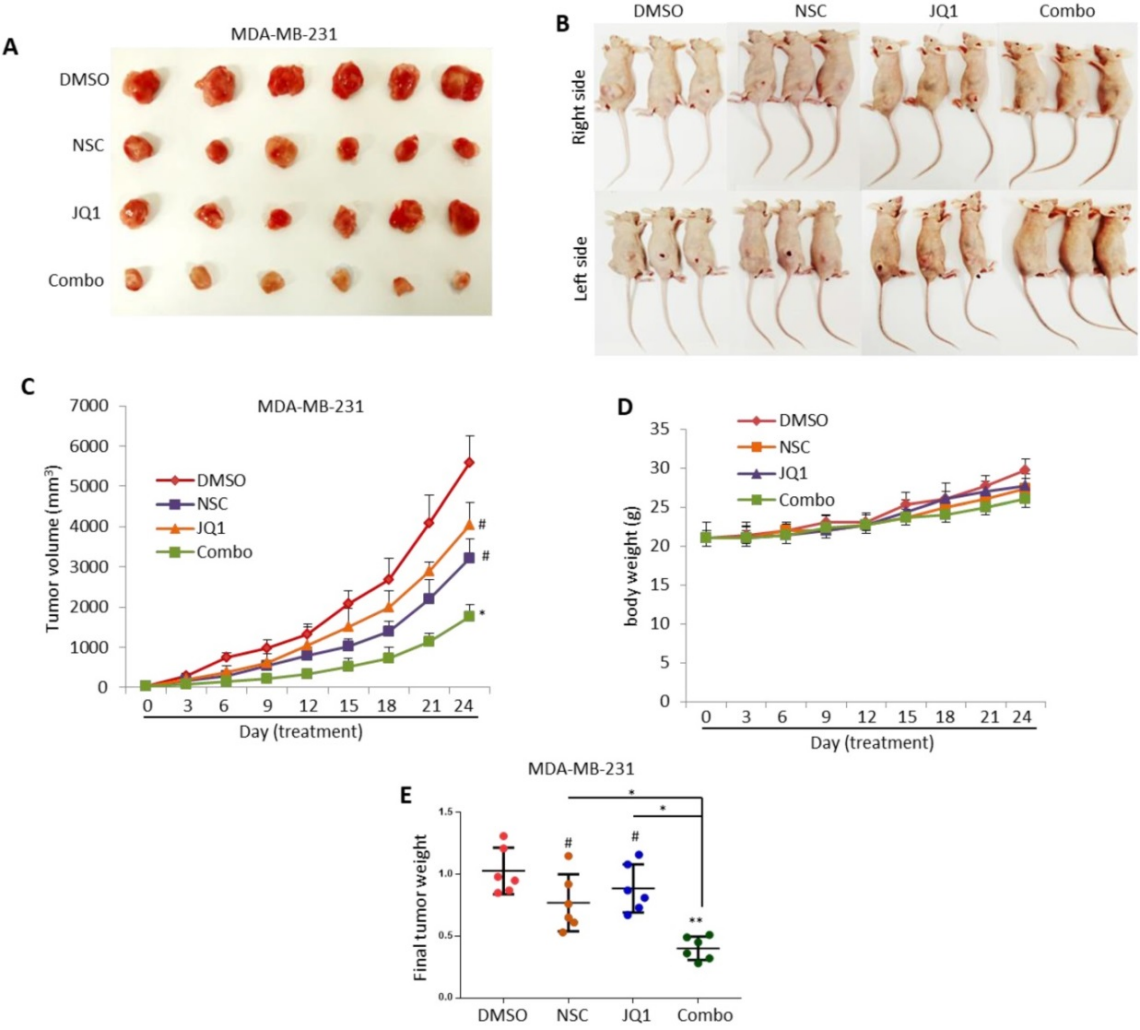
** NSC and JQ1 combined treatment suppresses MDA-MB-231 xenograft tumor growth. (A)** Mice bearing MDA-MB-231-based tumors were treated with DMSO, NSC23776, JQ1 and combination three times per week for three weeks. **(B)** Representative images of tumor-bearing treated and control mice were taken on the concluding day (day 24) of the experiment. **(C)** Xenograft tumor growth curves of tumor-bearing control and treated mice; tumor size was measured at days 0, 3, 6, 9, 12, 15, 18, 21and 24. **(D)** Body-weight of tumor-bearing control and treated mice were taken at days 0, 3, 6, 9, 12, 15, 18, 21 and 24. **(E)** Final xenograft tumor weight was taken on day 24 of the experiment. Student t-test was performed for tumor volume and tumor weight statistical analysis. *p < 0.05; **p < 0.005; ***p < 0.0005; # not significant.

The anti-growth effects of JQ1 and/or NSC treatment were also assessed *in vivo* using an MDA-MB-231 based xenograft tumor model. MDA-MB-231 breast cancer cells were transplanted into the right and left flank areas of female nude mice. Mice treated with JQ1 or NSC showed inhibited tumor growth in comparison with DMSO-treated control mice but did not result in significant anti-growth effects as assessed by tumor size or weight (Fig. 4A-C). In contrast, combination therapy resulted in a significant reduction in tumor growth relative to DMSO-treated control mice or mice treated with JQ1 or NSC (Fig. 4A-C). No significant body-weight loss was observed in the DMSO, JQ1 or NSC-treated mice, a slight decrease in body weight was observed in mice treated with JQ1+NSC (Fig. 4D). Final tumor weight analysis showed a significant reduction in tumor weight in mice treated with JQ1+NSC combination relative to control mice treated with DMSO (Fig. 4E). Taken together, these results suggest that JQ1+NSC combination therapy exerts significant anti-cancer effects in breast tumorigenesis and highlights the importance of combined inhibition of BRD4-RAC1 signaling in breast cancer development and progression.

### Combined NSC+JQ1 treatment targets c-MYC-G9a-FTH1 axis, induces autophagy and inhibits HDCA1 expression

**Figure 5:**
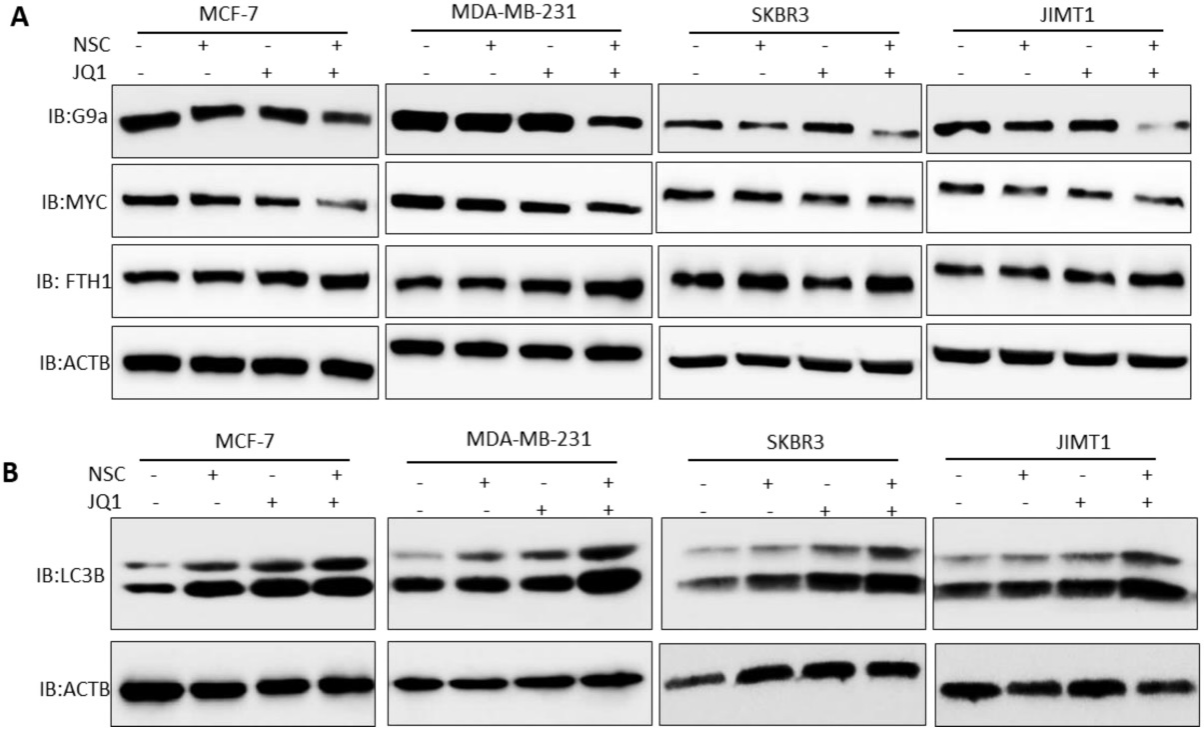
** Co-targeting BRD4 and RAC1 targets c-MYC-G9a-FTH1 axis and induces autophagy.** The expression of c-MYC, G9a and FTH1 was examined in MCF-7, MDA-MB- 231, SKBR3 and JIMT-1 cells treated with (**A**) JQ1 (2 uM) and/or NSC (30 uM) or combination for 72 h; β-actin was used as a negative loading control. Expression status of (**B**) LC3BI/II was evaluated in MCF-7, MDA-MB-231, SKBR3 and/or JIMT-1 cells at 72 h following treatment with JQ1 (2 uM) and/or NSC (30 uM) for 72 h. β-actin was used as a negative loading control.

To investigate the mechanism through which the co-inhibition of RAC1 and BRD4 precipitates such strong anti-growth effects in BRCA, the expression of key oncogenes and tumor suppressor genes under the control of or related to RAC1 and/or BRD4 signaling was assessed in control and treated cells. MCF-7, MDA-MB-231, SKBR3 and JIMT1 cells treated with “2 µM JQ1 + 30 µM NSC” showed reduced expression of G9a expression (Fig. 5A). Additionally, MDA-MB-231, SKBR3, JIMT1 and MCF-7 cells treated with “2 µM JQ1+ 30 µM NSC” showed a significant decrease in c-MYC expression and a significant increase in FTH1 expression (Fig. 5A). Next, we investigated protein-protein interactions between c-MYC, G9a and FTH1 to test whether these proteins form complex in breast cancer cells. A Co-immunoprecipitation (Co-IP) experiment was performed using the c-MYC antibody. Co-IP experiment showed a strong interaction between c-Myc and G9a as compared with IgG control (Fig. S3A). However, no interaction between between c-Myc and FTH1 or G9a and FTH1 was evident (Fig. S3A). This suggests that c-Myc and G9a may form complexes that may target and alter FTH1 expression independent of direct protein-protein interactions. Moreover, we tested the expression pattern of FTH1 in different molecular subtypes of breast cancer. We observed high expression of FTH1 in JIMT1 HER-2 positive breast cancer cells as compared with other breast cancer subtypes. The expression of FTH1 in MCF-7, MDA-MB-231 and BT549 breast cancer cells was lower than that in SKBR3, JIMT-1 and MDA-MB-468 (Fig. S3B). Inhibition of the expression of both c-Myc and G9a and consequent up-regulation of FTH1 expression points to a novel molecular mechanism that ensues following treatment with NSC and JQ1. This merits further investigation of the anti-tumor effects of this combination in breast cancer, and possibly other types of cancers.

**Figure 6:**
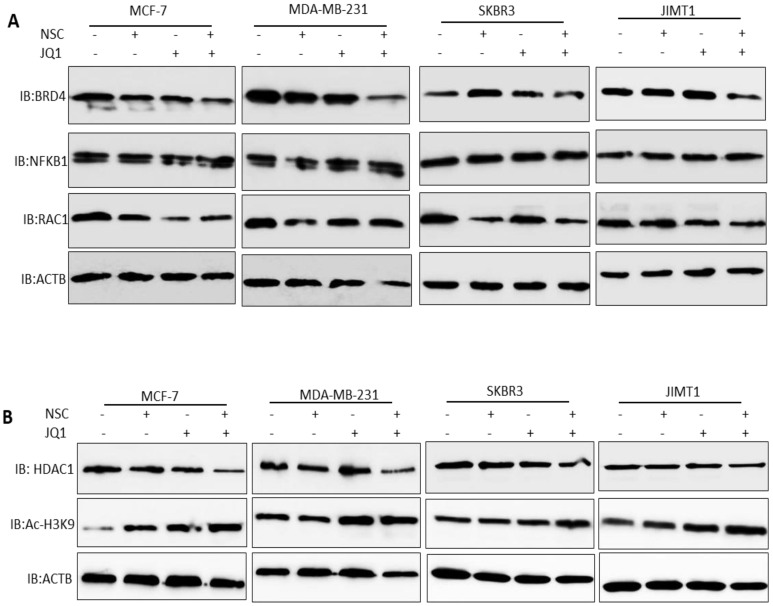
** Effects of JQ1 and NSC treatment on the expression of key cellular signaling pathways and HDAC1/Ac-H3K9 axis. (A)** The expression of BRD4, RAC1 and NFKB1 were examined in MCF-7, MDA-MB- 231, SKBR3 and JIMT-1 cells treated with JQ1 (2 µM) and/or NSC (30 µM) or combination for 72 h; β-actin was used as a negative loading control. **(B)** The expression of HDAC1 and AC-H3K9 were determined in MCF-7, MDA-MB- 231, SKBR3 and JIMT-1 cells treated with JQ1 (2 µM) and/or NSC (30 µM) or combination for 72 h; β-actin was used as a negative loading control.

Treatment with JQ1+NSC also resulted in increased expression and processing of the autophagosome marker LC3B (Fig. 5B), thus suggesting that this combination induces autophagy in different molecular subtypes of breast cancer. Moreover, we tested the expressions of NFKB1, RAC1and BRD4 in different molecular subtypes of breast cancer cells followed by NSC/JQ1 combined treatment. We observed a reduction in RAC1 and BRD4 expressions in combination treated cells in comparison to control cell lines treated with DMSO in MCF-7, MDA-MB-231, SKBR3 and JIMT-1 breast cancer cells (Fig. 6A). As expected, the expression of RAC1 and BRD4 was significantly inhibited in cells treated with the respective inhibitor or with the NSC+JQ1 combination (Fig. 6A). We detected a slight increase in NFKB1 expression in combined treated cells in comparison to DMSO.

Next, we tested the expression of HDAC1 and AC-H3K9 in control and combination treated cells. We detected a significant decrease in HDAC1 expression in MCF-7, MDA-MB-231, SKBR3 and JIMT-1 breast cancer cells that were treated with NSC/JQ1 combination as compared to control or single treatment (Fig. 6B), thus suggesting a novel role of this combination to by targeting HDAC1 expression to suppress breast cancer cells growth. In contrast, we observed increase in AC-H3K9 expression in JQ1/NSC combination treated cells, thus suggesting a role of this combination in histone modification and chromatin modeling (Fig. 6B). The expression profile of several additional markers related to cellular bioenergetics was also examined. As shown in the Supplementary Figures S4A and S4B, no consistent or significant pattern of change was discernable in any of the pathways investigated. Taken together, these results suggest that NSC/JQ1 combination suppresses breast cancer cells growth and tumorigenesis by targeting multiple signaling pathways including induction of autophagy via LC3B, disruption of c-MYC-G9a-FTH1 axis, modulation of histone modification and chromatin remodeling by targeting HDAC1/Ac-H3K9 axis in different molecular subtypes of breast cancer. These results points towards the novel and potential role of this therapeutic combination to target diverse cellular signaling pathways to suppress breast cancer progression and development.

### c-MYC silencing enhances the anti-tumor effects of JQ1+NSC treatment in different molecular subtypes of BRCA cells

**Figure 7:**
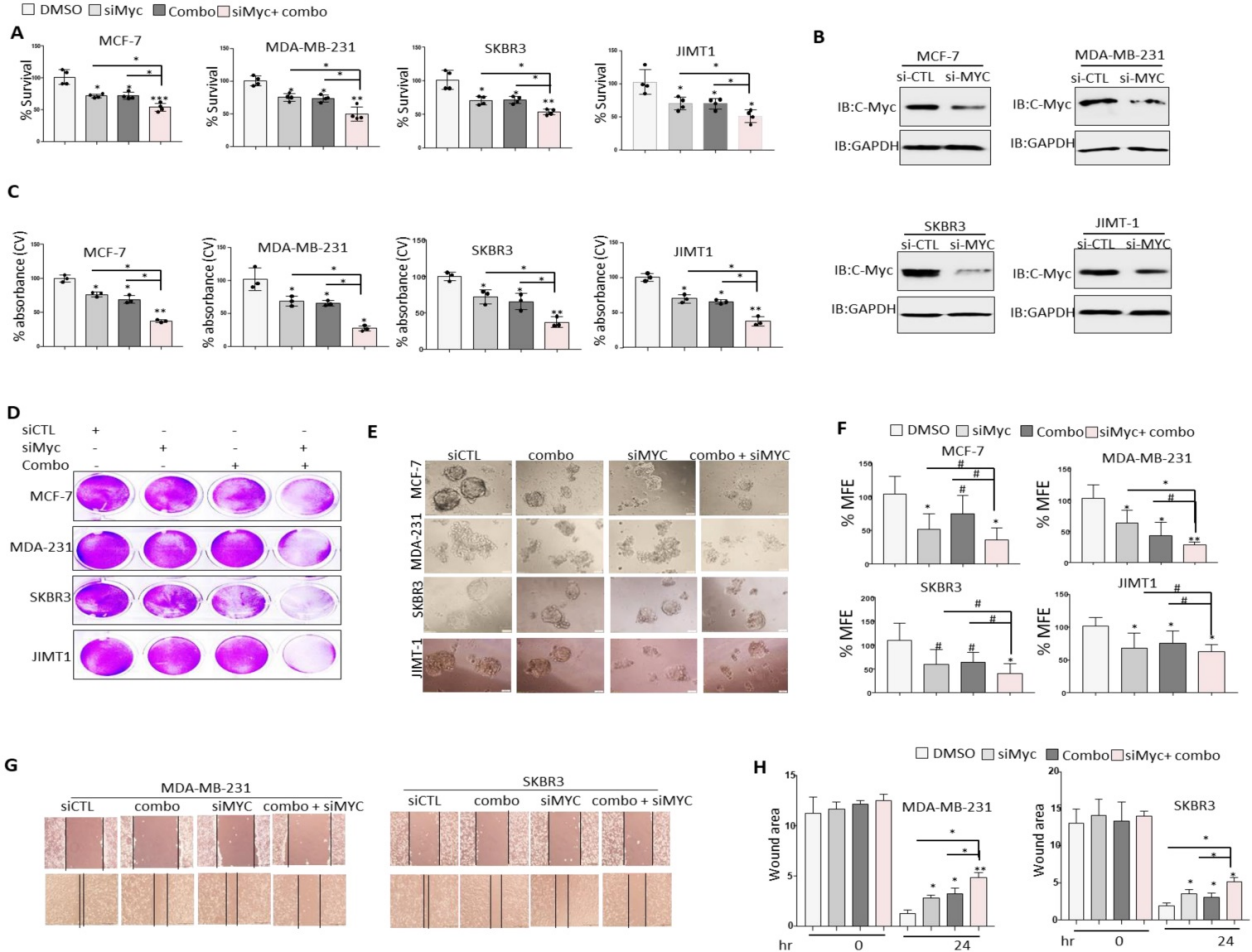
** Silencing of c-MYC further sensitizes BRCA cells to the anti-growth effects of JQ1 plus NSC treatment.** Cell viability as determined by (**A**) MTT assay in MCF-7, MDA-MB-231 and JIMT-1 cells transfected with control siRNA or c-MYC siRNA and treated with JQ1 (0.5 uM) and/or NSC (15 uM) for 72 h. *p < 0.05; **p < 0.005; ***p < 0.0005; # not significant. **(B)** Knockdown efficiency of c-MYC in MCF-7, MDA-MB-231 and JIMT-1 cells transfected with control siRNA or c-MYC siRNA for 72 hr. **(C)** Mean ±SD of crystal violet absorbance values as calculated based on three independent experiments. p < 0.05; **p < 0.005; ***p < 0.0005; # not significant. **(D)** Crystal violet staining data in MCF-7, MDA-MB-231 and JIMT-1 cells transfected with control siRNA or c-MYC siRNA and treated with JQ1 (0.5 µM) and/or NSC (15 µM) for 72 h. **(E)** Mammosphere formation in MCF-7, MDA-MB-231 and JIMT-1 cultures transfected with control or c-MYC siRNA and treated with JQ1 (0.5 µM) and/or NSC (15 uM) for 7 days. Data is representative of three separate experiments. **(F)** Percentage mammosphere forming efficiency (%MFE ± SD) as calculated based on three separate experiments. p < 0.05; **p < 0.005; ***p < 0.0005; # not significant. **(G)** MDA-MB-231 and SKBR3 transfected with control or c-MYC siRNA and treated with JQ1 (0.5 µM) and/or NSC (15 µM) for 48 h prior to performing the wound healing assay. Wound healing area was measured at 24 h post scratching; images shown are representative of three independent experiments. **(H)** Quantification of wound healing data from G. Data represents mean ± SD of wound healing potential based on three independent experiments. *p < 0.05; **p < 0.005; ***p < 0.0005; # no significant difference.

It is well established that c-MYC plays a pivotal role in chemoresistance and that silencing of c-MYC enhances chemosensitivity in cancer cells 12. Given that treatment of BRCA cells with JQ1+NSC is associated with a significant reduction in c-MYC expression, the question of whether reduced c-MYC expression prior to treatment with JQ1 and/or NSC could further sensitize target cells to the anti-growth effects of these drugs was examined. c-MYC knockdown reduced cell viability in comparison to control siRNA treated cells as shown by MTT (Fig. 7A). Interestingly, treatment of c-MYC-silenced cells with JQ1 and/or NSC resulted in noticeably greater levels of growth inhibition, especially at “0.5 µM JQ1 + 15 µM NSC” in MCF-7, MDA-MB-231, SKBR3 and JIMT-1 cells relative to similarly treated siRNA controls (Fig. 7A). MYC was knockdown in different molecular subtypes of breast cancer cells including MCF-7, MDA-MB-231, SKBR3 and JIMT1 (Fig. 7B). Crystal violet staining showed results consentient with MTT assay (Figs. 7C and 7D). Moreover, combined JQ1+NSC treatment of c-MYC-silenced cells also resulted in a significant reduction in mammosphere formation and inhibition of CSC-like expansion in comparison to control cells treated with DMSO in MCF-7 MDA-MB-231, SKBR3 and JIMT1 cells (Figs. 7E and 7F). Next, we determined the effect of JQ1+NSC combination therapy on MDA-MB-231 and SKBR3 cell migration following c-MYC silencing. Interestingly, c-MYC-silenced MDA-MB-231 and SKBR3 cells treated with JQ1+NSC showed a further reduction in cell migration potential relative to control cell lines (Figs. 7G and 7H). Taken together, these results suggest an important role of NSC+JQ1 combination in c-MYC silenced cells to further reduce cell growth, cancer stem cells formation and cell migration in different molecular subtypes of breast cancer and describe a role of decrease expression of c-MYC in sensitization of breast cancer cells to combined treatment.

### Pre-treatment with vitamin C further sensitizes different molecular subtypes of BRCA cells to NSC/JQ1 combination

**Figure 8:**
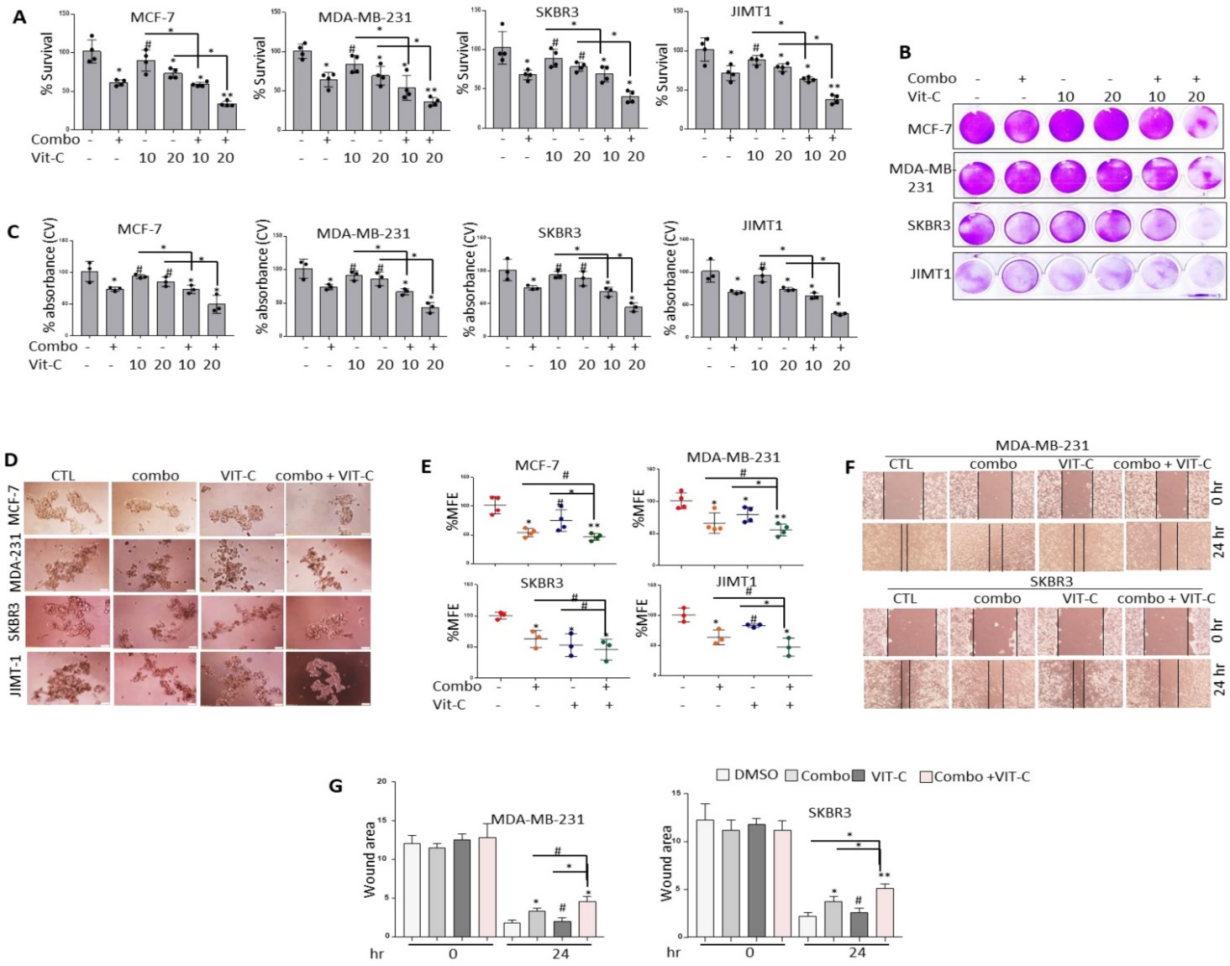
** Vitamin C further sensitizes BRCA cells to the anti-growth effects of JQ1 plus NSC treatment.** Cell viability as determined by (**A**) MTT assay or (**B**) Crystal violet staining in MCF-7, MDA-MB-231, SKBR3 and JIMT-1 cells treated with vitamin C (10 or 20 uM), JQ1 (0.5 uM) and NSC (15 uM) for 72 h. **(C)** Mean ±SD of crystal violet absorbance values as calculated based on three independent experiments. p < 0.05; **p < 0.005; ***p < 0.0005; # not significant. **(D)** Mammosphere formation in MCF-7, MDA-MB-231 and JIMT-1 cultures treated with vitamin C, JQ1 (0.5 uM) and NSC (15 uM) for 7 days. **(E)** Percentage mammosphere forming efficiency (%MFE ± SD) as calculated based on three separate experiments. p < 0.05; **p < 0.005; ***p < 0.0005; # not significant. **(F)** MDA-MB-231 and SKBR3 treated with vitamin C (10 or 20 µM) ± JQ1 (0.5 µM) and NSC (15 µM) for 48 h prior to performing the wound healing assay. Wound healing area was measured at 24 h post scratching; images shown are representative of three independent experiments. **(G)** Quantification of wound healing data from F. Data represents mean ± SD of wound healing potential based on three independent experiments. *p < 0.05; **p < 0.005; ***p < 0.0005; # no significant difference.

Vitamin C has been shown to promote apoptosis, suppress cancer cell proliferation 41 and enhance cancer immunotherapy 42. Moreover, combined JQ1 plus vitamin C was previously reported to induce apoptosis in TNBC and melanoma cells 43,44. Based on these observations, the combined effects of vitamin C and JQ1+NSC treatment on cancer cell viability and CSC-like expansion was examined in MCF-7, MDA-MB-231, SKBR3 and JIMT1 cells. Combined vitamin C plus JQ1+NSC treatment inhibited cell viability at significantly higher levels relative to that in cells treated with JQ1 and/or NSC alone (Fig. 8A-C). Furthermore, significant inhibition of CSC-like formation was noted in MCF-7, MDA-MB-231, SKBR3 and JIMT1 cells treated with vitamin C plus JQ1+NSC (Fig. 8D-E). Next, vitamin C plus JQ1+NSC treatment further inhibited cell migration of MDA-MB-231 and JIMT1 cells relative to untreated controls, VIT-C or JQ1+NSC treated cells (Fig. 8F-G). Taken together, these results suggest that combination of NSC/JQ1 with VIT-C further suppresses cell growth, cancer stem cells formation and cell migration of breast cancer cells and describe a role of vitamin C in sensitization of different molecular subtypes of breast cancer to combined treatment.

### Co-targeting RAC1 and BRD4 does not induce ferroptosis

**Figure 9:**
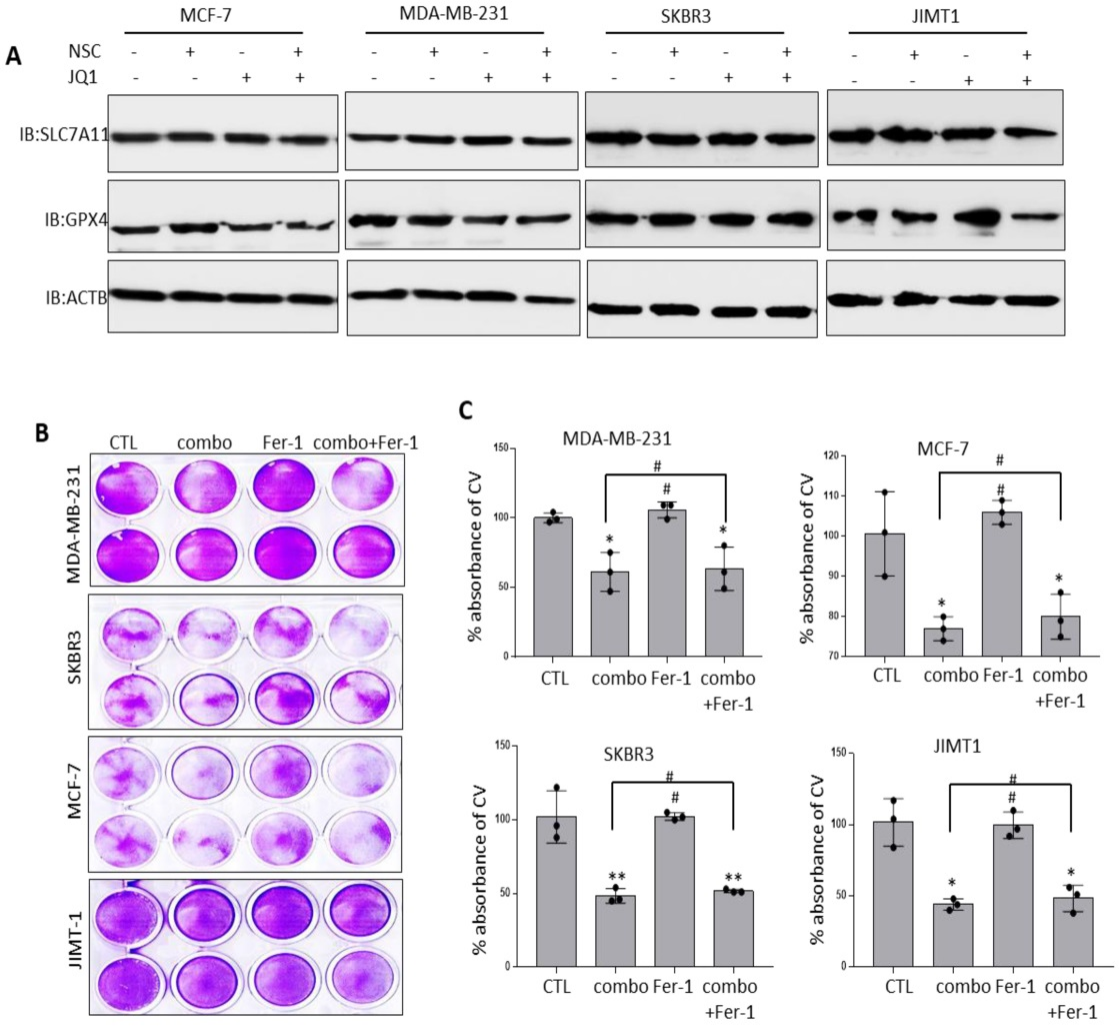
** Combined JQ1 plus NSC treatment does not promote ferroptosis in BRCA cells. (A)** Expression of SLC7A11 and GPX4 proteins in MCF-7, MDA-MB-231, SKBR3 and JIMT-1 cells at 72 h -post-treatment with JQ1 (2 uM) and/or NSC (30 uM) for 72 h; β-actin was used as a negative loading control. **(B)** Cell viability as determined by crystal violet staining in MCF-7, MDA-MB-231, SKBR3 and JIMT-1 cells treated with Ferrostatin-1 (Fer-1) for 72 h in the presence/absence of JQ1 (2 uM) plus NSC (30 uM). **(C)** Mean ±SD of crystal violet absorbance values as calculated based on three independent experiments. *p < 0.05; **p < 0.005; ***p < 0.0005; # no significant difference.

The observation that co-targeting RAC1 and BRD4 associated with increased FTH1 expression raised the possibility that this approach may disrupt cellular iron metabolism and induce ferroptosis. Previous work has suggested that altered expression of key negative regulators of ferroptosis including SLC7A11 and GPX4 has a significant bearing on cancer progression 45. To examine this possibility, the expression of SLC7A11 and GPX4 was assessed in BRCA cells treated with JQ1 and/or NSC. As shown in (Fig. 9A), treatment with JQ1/NSC combination resulted in reduced expression of GPX4 in MCF-7 and JIMT-1 cells but not in MDA-MB-231 and SKBR3 cell lines. Although treatment with JQ1 resulted in reduce expression of GPX4 in MDA-MB-231 cells, but combined treatment increased its expression (Fig. 9A). GPX4 expression did not change in SKBR3 cells following treatment with JQ1 and/or NSC. Additionally, we did not observed any significant changes in SLC7A11 expression in NSC/JQ1 alone or combination treatment in breast cancer cells (Fig. 9A). Given that differential expression of NOX4 and other ferroptosis-related genes may reflect variations in the genetic background of the different cells used, further examination of the potential of JQ1 and/or NSC to induce ferroptosis was carried out by treating cells with the ferroptosis inhibitor ferrostatin-1 (Fer-1). Fer-1 treatment did not enhance growth in control cells and did not reverse the growth inhibitory effects of JQ1+NSC in any of the cell lines tested (Fig. 9B-C). This raised the possibility that JQ1 and NSC may induce cytostatic rather than cytotoxic effects.

### Clinicopathalogical analysis of RAC1 and BRD4 expressions in different molecular subtypes of breast cancer samples

**Figure 10:**
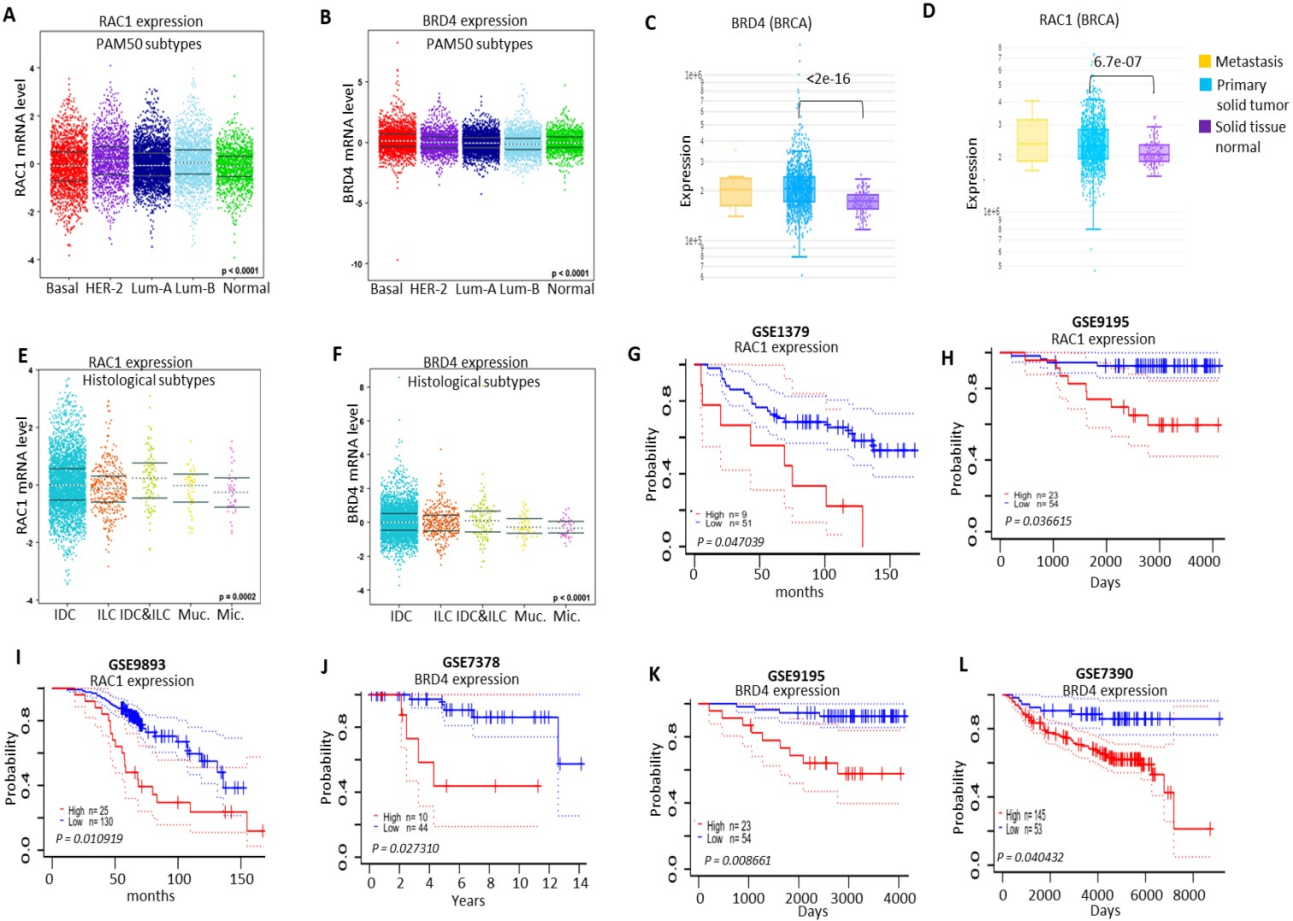
** Clinicopathalogical significance of RAC1 and BRD4 in BRCA. (A and B)** Expression of RAC1 and BRD4 in basal-like, luminal-A, luminal-B and HER-2+ BRCA (PAM50 subtypes) molecular subtypes using the Breast Cancer Gene-Expression Miner v4.4 database. **(C and D)** Comparison of RAC1 and BRD4 expression in primary solid breast tumors vs normal solid breast tissue. Data was generated using DriverDBv3 database 63. **(E and F)** RAC1 and BRD4 expression profile in histological subtypes of breast cancer including Invasive ductal carcinoma (IDC), Invasive lobular breast cancer (ILC), mucinous breast cancer (Muc) and micropapillary breast cancer (Mic.). This data was generated using the Breast Cancer Gene-Expression Miner v4.4 database. **(G-I)** Survival analysis of RAC1 high and low expression in BRCA patients samples using prognoscan database. **(J-L)** Survival analysis of BRD4 high and low expression in BRCA patients samples using prognoscan database.

Next, we analyzed the expression pattern of RAC1 and BRD4 in different molecular subtypes of breast cancer. We observed higher expression levels of both RAC1 and BRD4 in different molecular subtypes of breast cancer in comparison to normal-like breast samples (Figs. 10A and 10B; S5A and S5B). We also compared the expression levels of RAC1 and BRD4 in primary solid breast tumors relative to normal solid tissues. High expression levels of both RAC1 and BRD4 were observed in primary solid breast tumors (Figs. 10C and 10D). We also analyzed the expression levels of both RAC1 and BRD4 in different histological subtypes of breast cancer and nodal status. We observed a significant expression pattern of both RAC1 and BRD4 in different hisotological subtypes (Figs. 10E and 10F). Morover, high expression of RAC1 but not BRD4 correlates with nodal status (Figs. S5C and S5D). We also detected a correlation of RAC1 and BRD4 expressions with leukocyte infiltration and somatic copy number alteration (SCNA) in breast cancer samples (Figs. S6A, S6B and S7A, S7B).

Based on high expression of both RAC1 and BRD4 in human breast cancer samples, we investigated the effect of high and low expression levels of RAC1 and BRD4 on survival of breast cancer patients. RAC1 analysis from breast cancer datasets GSE1379, GSE9195 and GSE9893 showed that high expression of RAC1 predicts poor survival of breast cancer patients. In contrast, lower expression of RAC1 was correlated with better survival of breast cancer patients (Figs. 10G-I). Moreover, analysis of BRD4 expression from GSE7378, GSE9195 and GSE7390 revealed that lower expression of BRD4 predicts prolong survival of breast cancer patients in comparison to high expression of BRD4 (Figs. 10J-L). Taken together, these results suggest that different molecular subtypes of breast cancer express high levels of RAC1 and BRD4 and amplification of both BRD4 and RAC1 signaling correlated with decreased survival of breast cancer patients.

## Discussion

In the present study, we showed a novel co-targeting strategy by combined inhibition of BRD4-RAC1 signaling pathways in different molecular subtypes of breast cancer including luminal-A, HER-2 positive and triple-negative breast cancer (TNBC). Our data suggest that combined inhibition of BRD4-RAC1 pathways represents a potential therapeutic approach in different molecular subtypes of breast cancer and highlights the importance of RAC1-BRD4 signaling in breast tumorigenesis and development by targeting disruption of C-MYC/G9a/FTH1 axis and down regulation of HDAC1. The heterogeneous nature of BRCA has proven to be a persistent obstacle in the development of effective targeted therapies against the disease. Previous work has shown that therapeutic strategies that target more than one metabolic process or signaling pathway could lessen the potential of cancer cells to develop resistance 46. This, on top of additional potential advantages including better efficacy, dose reduction and minimal toxicity 47,48.

Combination therapies involving an alkylating agent such as cyclophosphamide and an anti-metabolite such as methotrexate or 5-fluorouracil were previously discussed as potential therapeutic approaches in BRCA 49. As described previously, BRD4 and RAC1 oncoproteins play important roles in cancer cell growth, migration, invasion and metastasis, hence the focus on these two oncogenes as potential therapeutic targets in different cancers 23,50,51. BRD4, which binds to acetylated lysine (KAc) residues on histone tails to regulate chromatin structure and gene expression, has been shown to occupy super-enhancer regions of multiple oncogenic proteins 12,51. BRD4 amplification has been associated with cancer cell growth, invasion and metastasis through transcriptional activation of the c-MYC oncogene 9. Several BET bromodomain inhibitors that disrupt BRD4-dependent transcriptional activation are currently available as anti-cancer drug candidates 9,12,51. RAC1, a key regulator of multiple oncogenic pathways including mTORC1/2 and PAK1/2/3 among others 22 is emerging as a significant therapeutic target in different cancers 20.

In the present study, we showed that the expression of RAC1 and BRD4 is positively correlated in BRCA. We also showed that the expression of RAC1 and BRD4 differentially correlate with the level of infiltration of different leukocyte subsets in BRCA and that high expression levels of RAC1 and BRD4 associate with poor prognosis and reduced survival. These findings along with a number of previous studies which addressed the oncogenic roles of these two oncogenes 7-26 justified further examination of the anti-growth effects of a combination therapy that co-targets them concurrently. Co-targeting BRD4 and RAC1 precipitated very significant anti-growth effects in BRCA cells *in vitro* and inhibited tumor growth *in vivo*. This is based on the observation that treatment of BRCA cells with JQ1+NSC suppressed cell growth, migration and CSC-like expansion, induced cell senescence and autophagy, reduced the expression of key oncogenes and increased the expression of FTH1. Additionally, co-inhibition of RAC1 and BRD4 by JQ1+NSC treatment reduced MDA-MB-231-based tumor growth *in vivo*.

Mechanistically, our study showed that combined NSC+JQ1 treatment inhibited BRCA cell growth by down regulating the expression of key oncogenes including G9a and c-MYC along with HDAC1, BRD4 and RAC1. RAC1 is a key regulator of multiple oncogenic pathways including activation of mTORC1/2, PAK1/2/3 and MAPK signaling, which plays an important and critical role in cancer metastasis and cell survival. Moreover, the RAC1 signaling pathway plays a central role in angiogenesis, cancer metastasis, tumor development, chemoresistances and invasion. Knockdown of RAC1 or inhibition of its expression was previously linked to decreased tumor cell growth, invasion and chemoresistance. Thus, RAC1 signaling pathway is considered an important and potential therapeutic target in different types of cancer 20. Expression of G9a, a nuclear histone lysine methyltransferase (HMT) that catalyzes H3K9 methylation, was previously reported to upregulate in different cancers and to play important roles in cancer invasion and migration 19.

Increased expression of c-MYC has been amply shown to promote metastasis of and resistance to chemotherapy in TNBC 52,53. Increased c-MYC expression was also reported to activate a network of oncogenic and MAPK signaling oncoproteins that promote cell survival 54. Moreover, silencing of c-MYC was reported to increase tumor sensitivity to chemotherapy 12. Consistent with these observations, silencing of c-MYC further enhanced the sensitivity of various breast cancer cell lines to the anti-growth effects of JQ1+NSC treatment. This observation is further supported by the finding that treatment of BRCA cells with JQ1+NSC associated with strong inhibition of HDAC1. Increased HDAC1 expression is known to induce resistance to chemotherapy in cancer cells by, for example, regulating the antioxidant system 55. Further evidence in support of the conclusion that JQ1+NSC treatment renders BRCA cells more sensitive to chemotherapy derives from the observation that JQ1+NSC-treated cells showed increased NOX4 expression. NOX4, the non-mitochondrial ROS-producing NADPH oxidase, which is highly expressed in BRCA 56 was previously reported to promote cancer cell proliferation, metastasis, transformation and to induce resistance to radiation 56-59.

The ability of JQ1+NSC treatment to reduce metastatic potential is further supported by the observation that this treatment approach reduced CSC-like potential 60 and the observation that it reduced the expression of the histone demethylase and stemness marker 61 lysine-specific demethylase 1 (LSD1). It is worth noting that co-targeting of RAC1 and BRD4 induced cytostatic rather than cytotoxic effects whether in the form of ferroptosis or apoptosis. This is consistent with the observation that this combined treatment approach up regulates FTH1 expression and reduces the potential for oxidative stress and ferritinophagy 13,25. FTH1 was previously reported to exert significant anti-tumor effects 33 by inhibiting the expression of oncogenic microRNAs 29, promoting EMT 30,31 and enhancing the transcriptional activity of p53 33 among other mechanisms. Increased FTH1 expression was also reported to render metastatic cancer more sensitive to chemotherapy 62.

In summary, our results showed a novel therapeutic strategy by co-targeting BET bromodomain BRD4 and RAC1 signaling pathways in different molecular subtypes of breast cancer including luminal-A, TNBC and HER-2 positive breast cancer. Functionally, combined inhibition of BRD4-RAC1 signaling pathways suppressed tumor growth in xenograft mouse model, inhibited the oncogenic potential, CSCs expansion and cell migration and induced cellular senescence and autophagy in different molecular subtypes of breast cancer. Mechanistically, combined inhibition of BRD4-RAC1 signaling pathways targeted c-MYC-G9a-FTH1 axis and revealed a novel molecular mechanism that might play important role in inhibition of breast cancer progression. Inhibition of HDAC1 and induction of Ac-H3-K9 expression by this combination suggest a role of this combined therapy in chromatin remodeling and histone modification. Depletion of c-MYC further sensitized different molecular subtypes of breast cancer cells to combined treatment, thus highlighting a key role of decreased c-MYC expression in drug sensitivity. Co-treatment with Vitamin C showed further sensitivity to combined treatment in different molecular subtypes of breast cancer. TCGA breast cancer samples showed a positive correlation of RAC1 with BRD4 and high expression of both proteins predicted poor prognosis and survival of breast cancer patients. Our results indicate that amplification of BRD4 and RAC1 dependent signaling pathways servers as independent biomarkers for breast cancer prognosis and furthermore these findings highlights towards the importance of combined inhibition of RAC1 and BRD4 signaling in different molecular subtypes of breast cancer. Taken together, our study identified a novel and potential therapeutic strategy by combined inhibition of BRD4-RAC1 signaling to suppress breast cancer development and tumorigenesis via c-MYC/G9a/FTH1 axis and down regulation of HDCA1 in different molecular subtypes in a context-dependent manner.

## Supplementary Material

Supplementary figures.

## Figures and Tables

**Figure 11:**
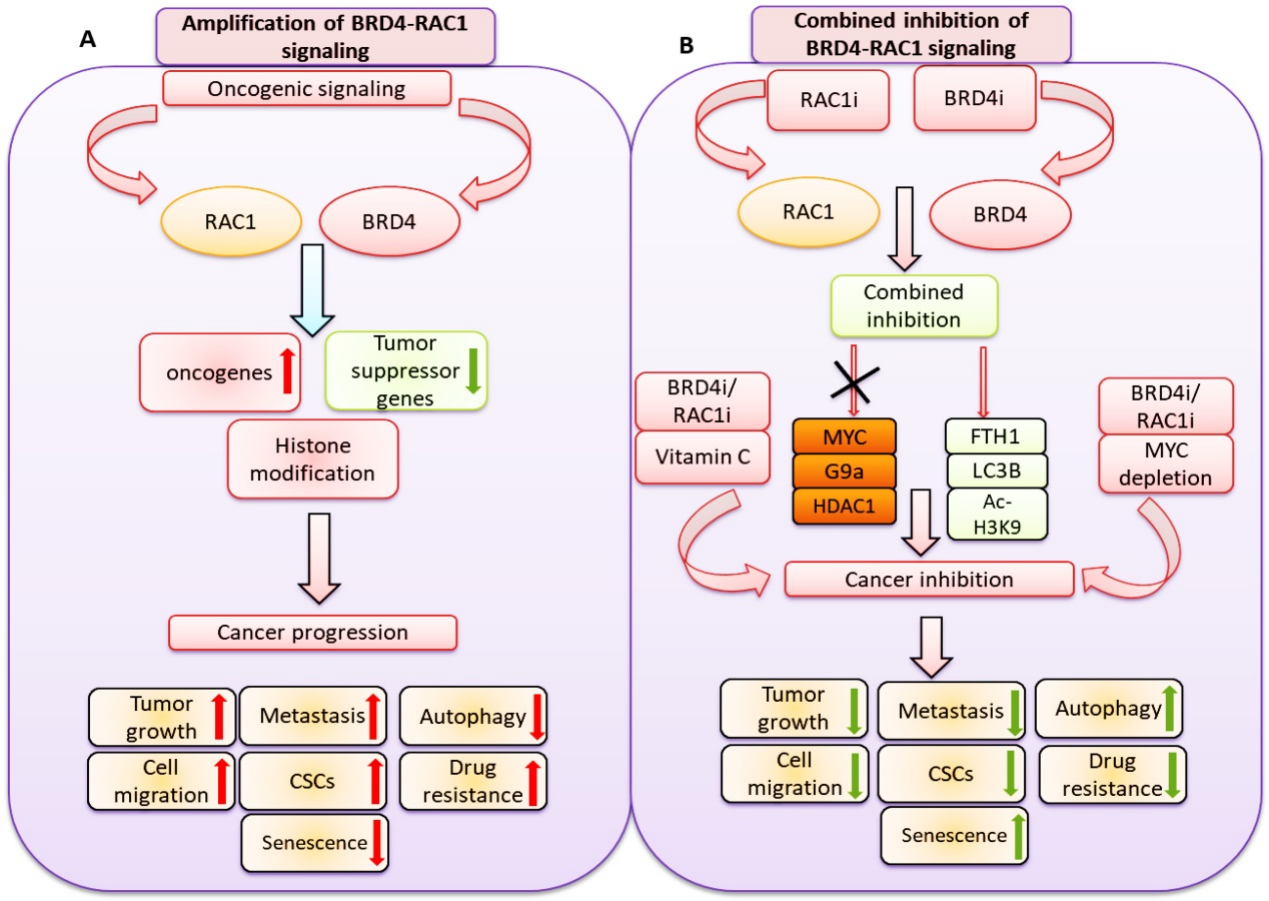
** A hypothetical model illustrating: A)** oncogenic signaling pathways promote BRD4 and RAC1 expressions and thereby activate oncogenic proteins, suppress tumor suppressor genes expression and target histone modification to facilitate cancer progression by targeting tumor growth, metastasis, cell migration, cancer stem cells (CSCs) expansion, drug resistance and autophagy. **B)** Combined inhibition of BRD4-RAC1 signaling pathways by BRD4 inhibitors (BRD4i) and RAC1 inhibitors (RAC1i) suppress c-MYC, G9a and HDAC1 oncogenic protein expressions and increase FTH1, LC3B and Ac-H3K9 levels to exert tumor suppressive effects. Combination of BRD4i and RACi coupled with c-MYC depletion or co-treatment of Vitamin C further suppress tumor growth, metastasis, cell migration, cancer stem cells (CSCs) expansion, drug resistance and induce autophagy.

**Table 1:** Drug combination index (CI)* in MCF-7, MDA-MB-231, JIMT1 and SKBR3 cells treated with different combinations of NSC23776 and JQ1

Cell line	NSC23776 + JQ1 combination (μM)			
	10.0 + 0.25	15.0 + 0.5	20.0 + 1.0	30.0 + 2.0
JIMT1	0.681**	0.727	0.758	0.387
MDA-MB-231	1.089	1.085	1.072	0.671
MCF-7	1.002	0.927	0.878	0.790
SKBR3	0.977	0.862	0.711	0.708

*CI was calculated based on three-five independent experiments using the Combosyn software (http://www.combosyn.com). ** *CI = 1* indicates additivity, *CI > 1* antagonism and *CI < 1* synergism.
