# Supplementary material for: Co-targeting BET bromodomain BRD4 and RAC1 suppresses growth, stemness and tumorigenesis by disrupting the c-MYC-G9a-FTH1axis and downregulating HDAC1 in molecular subtypes of breast cancer
Source: Int J Biol Sci. 2021 Oct 25;17(15):4474–92. doi: 10.7150/ijbs.62236 (PMC8579449; doi:10.7150/ijbs.62236)

**Supplementary figures**

**Figure S1:** Co-treatment with JQ1 plus NSC suppresses the growth of breast cancer cells. (A) MTT-based viability of BT549 (invasive ductal carcinoma), (B and C) Crystal violet staining-based assessment of viability in BT549 and MDA-MB-468 (invasive ductal carcinoma) treated with increasing concentrations of JQ1 and/or NSC for 72 h. (D and E) Mean ± SD of crystal violet absorbance values based on three independent experiments. *p < 0.05; **p < 0.005; ***p < 0.0005; # no significant difference.

**Figure S2:** Effect of JQ1 plus NSC combined treatment on primary cells. (A) AC16 (proliferating human cardiomyocyte, primary) and (B) 3T3 (mouse embryonic fibroblasts, primary) cells treated with increasing concentrations of JQ1 and/or NSC for 72 h. (C and D) Crystal violet staining-based assessment of viability in AC16 and 3T3 cells treated with increasing concentrations of JQ1 and/or NSC for 72 h. (E and F) Mean ± SD of crystal violet absorbance values based on three independent experiments. *p < 0.05; **p < 0.005; ***p < 0.0005; # no significant difference.

**Figure S3:** (A) Co-immunoprecipitation (CO-IP) of c-MYC with G9a and FTH1in MDA-MB-231 cells; an unrelated IgG antibody was used as a negative control. (B) Expression profile of FTH1 in different molecular subtypes of breast cancer including MCF-7, MDA-MB-231, MDA-MB-468, JIMT-1, SKBR3 and BT549.

**Figure S4:** Expression status of (A) LSD1 and GLUT4 expressions in MCF-7 and (B) NOX4 was evaluated in MCF-7 and MDA-MB-231 at 72 h following treatment with JQ1 (2 µM) and/or NSC (30 µM) for 72 h. β-actin was used as a negative loading control.

**Figure S5:** (A and B) Expression of RAC1 and BRD4 in basal-like, luminal-A, luminal-B and HER-2+ BRCA (Hu's subtypes) molecular subtypes. Data presented was generated using the Breast Cancer Gene-Expression Miner v4.4 database. (C and D) Nodal status of RAC1 and BRD4 in BRCA samples. Data presented was generated using the Breast Cancer Gene-Expression Miner v4.4 database.

**Figure S6:** RAC1 and BRD4 expression in relation to leukocyte tumor infiltration in BRCA. Correlation of (A) RAC1 or (B) BRD4 expression with level of infiltration of B cells, CD4+ T cells, CD8+ T cells, macrophage, neutrophil and dendritic cell in BRCA tissues; analysis was done 746 using the TCGA/TIMER database.

**Figure S7:** RAC1 and BRD4 somatic copy number alteration (SCNA) in relation to leukocyte infiltration in BRCA. Correlation of (A) RAC1 (B) BRD4 SCNA with level of infiltration of B cells, CD4+ T cells, CD8+ T cells, macrophage, neutrophil and dendritic cells; analysis was done using the TCGA/TIMER database.

# Supplementary figure S1

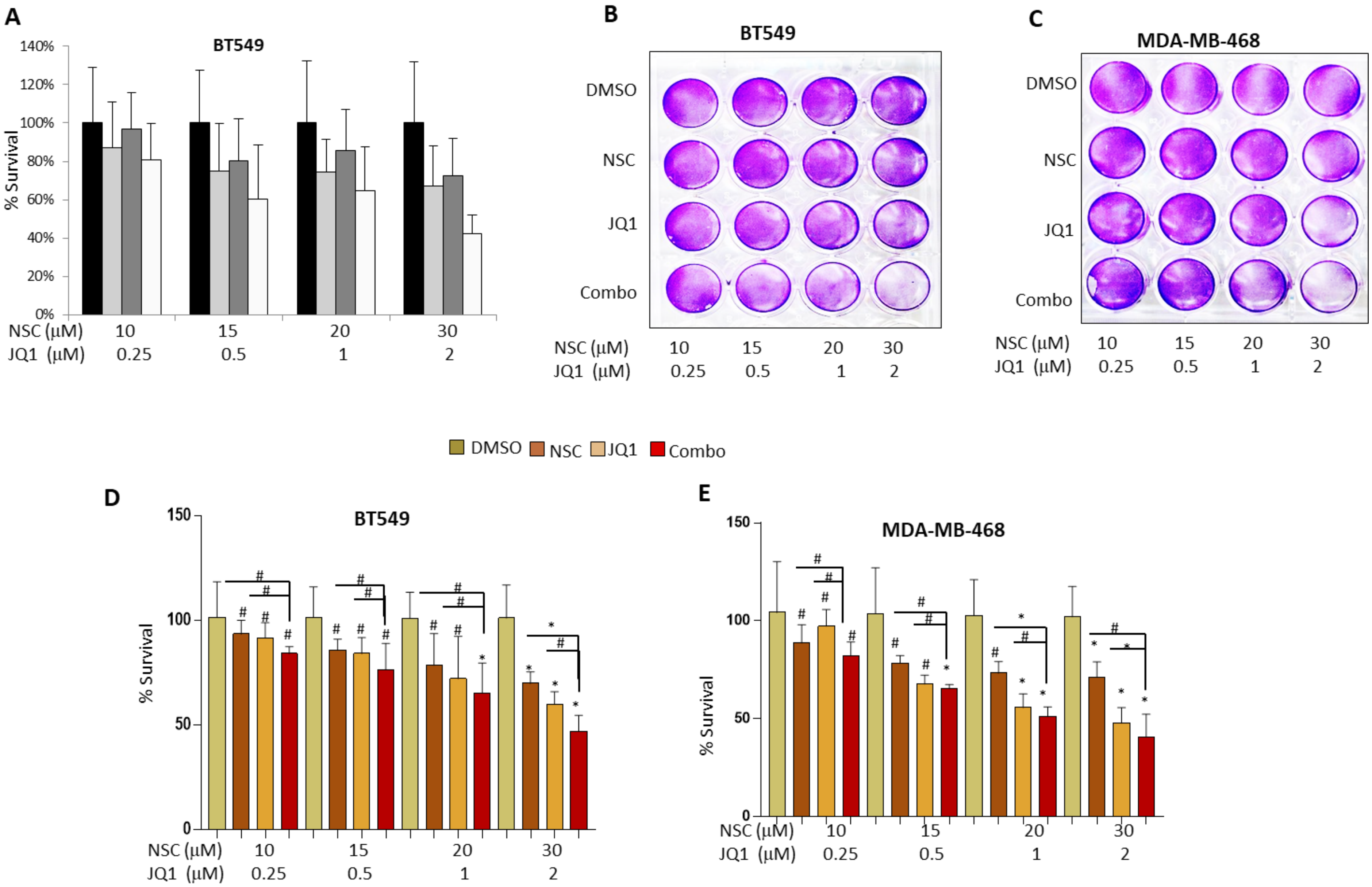

# Supplementary figure S2

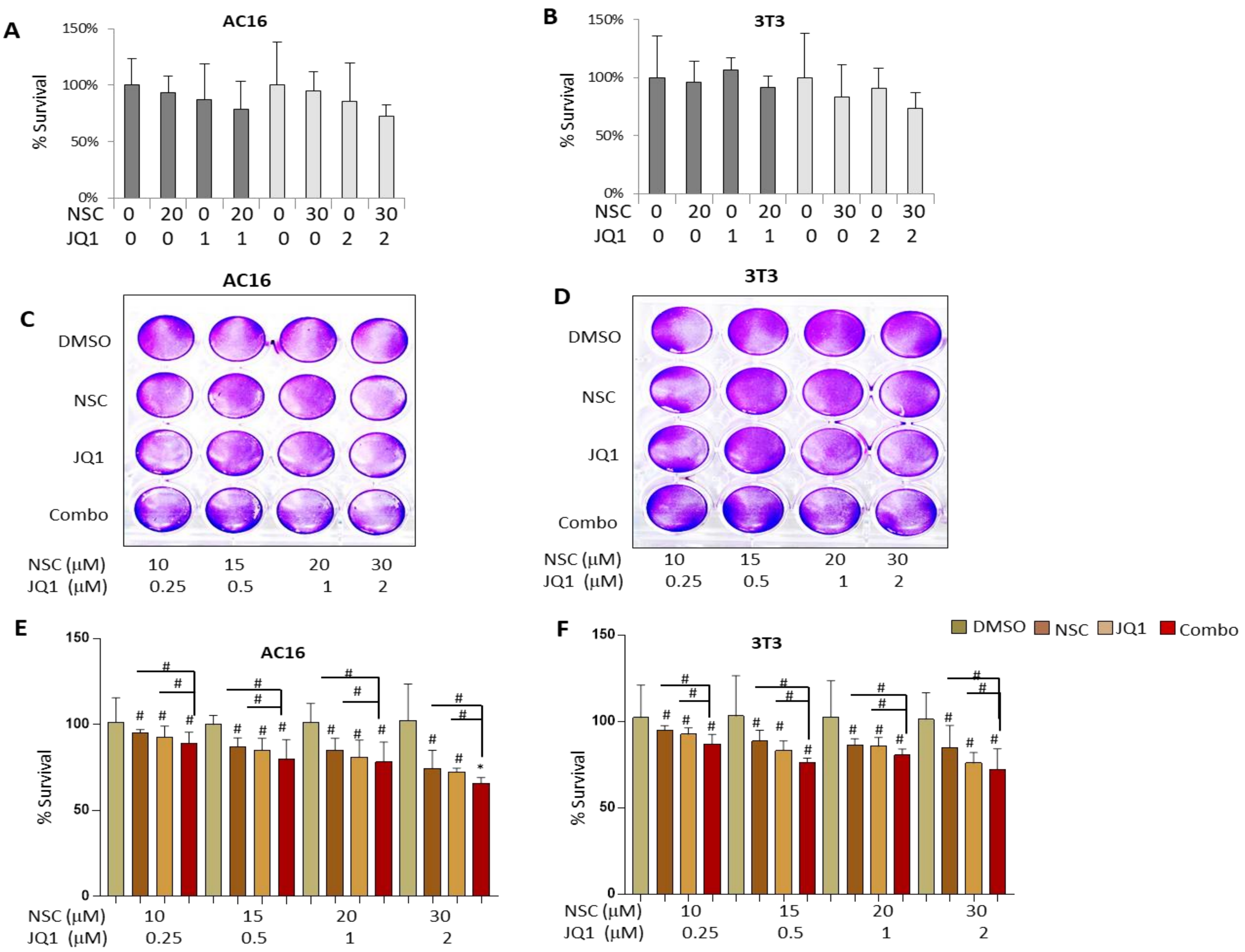

# Supplementary figure S3

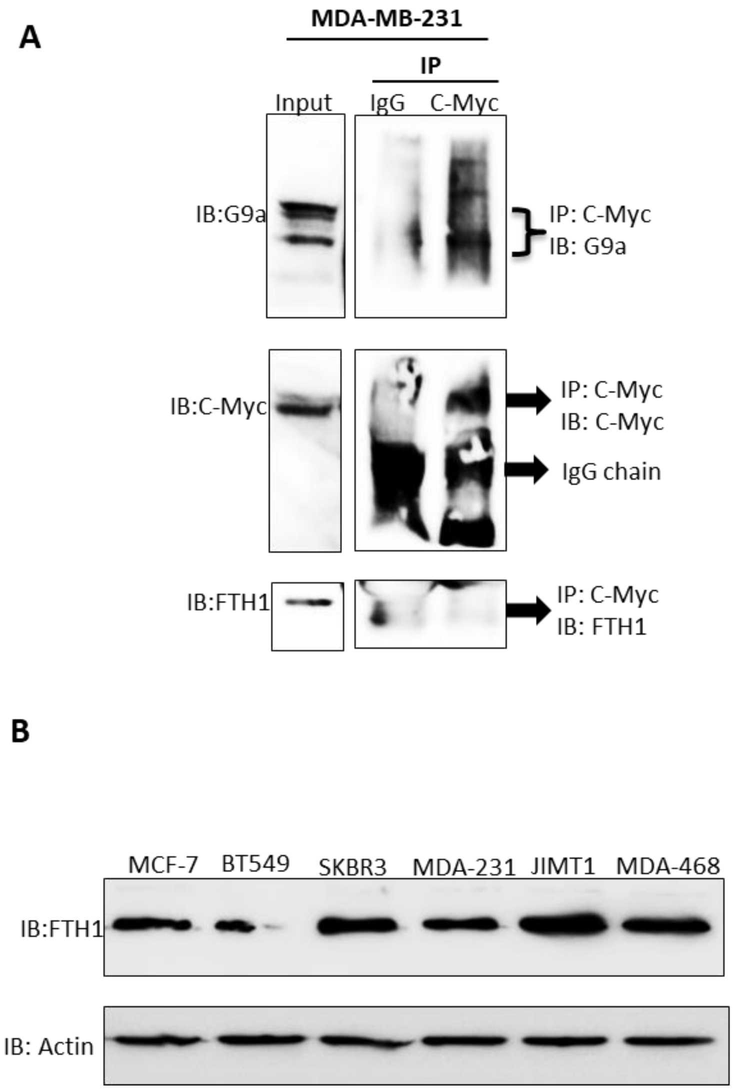

Supplementary figure S4

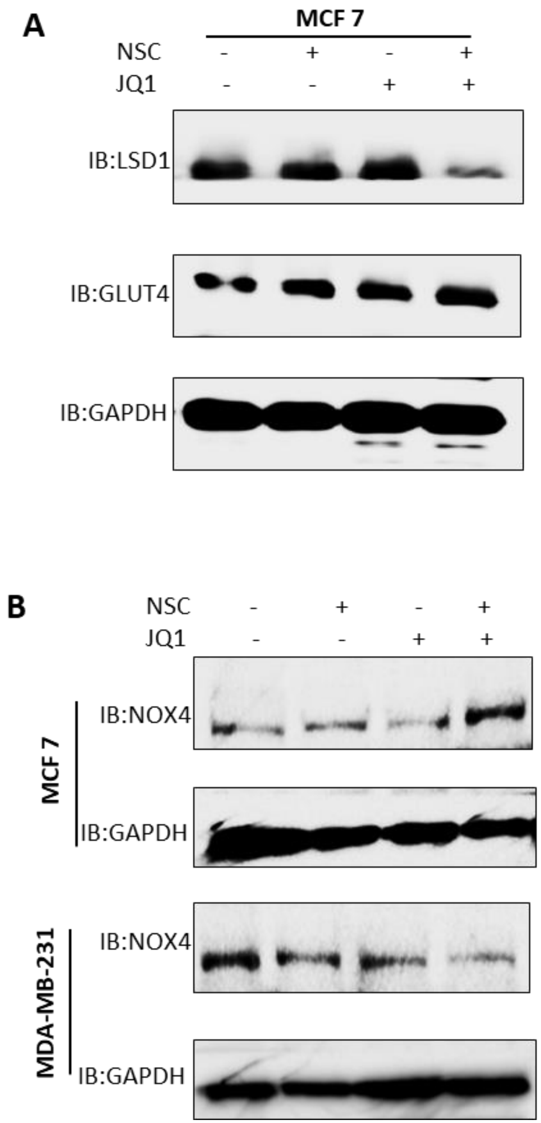

# Supplementary figure S5

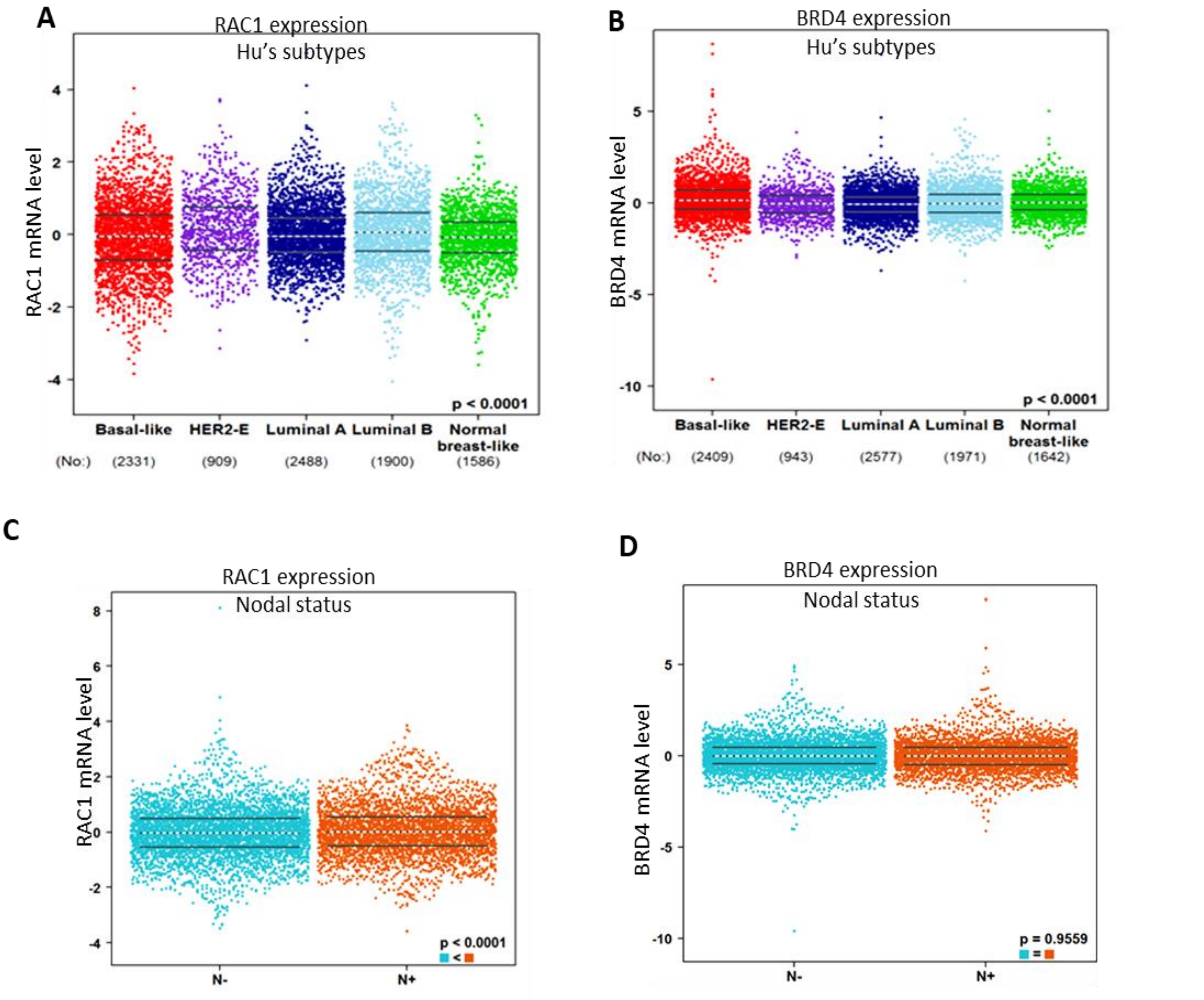

Supplementary figure S6

A

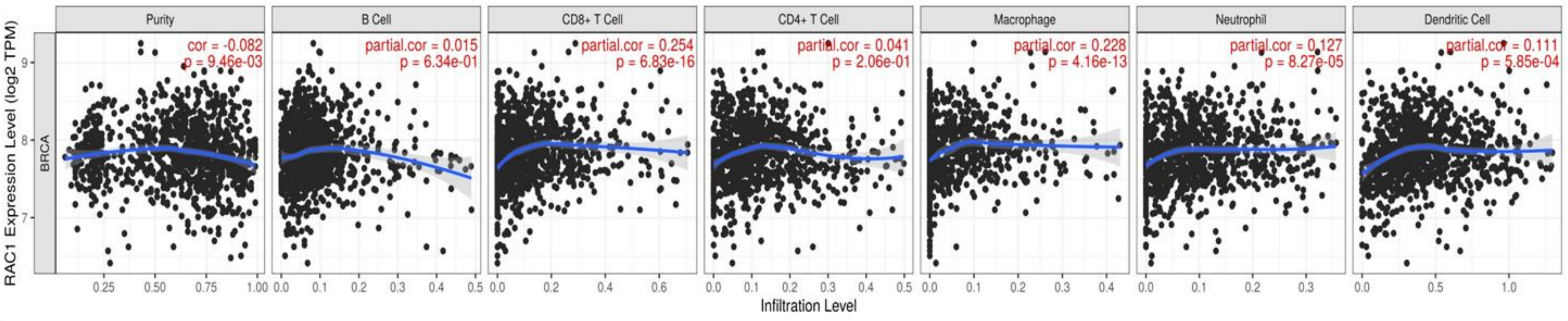


B

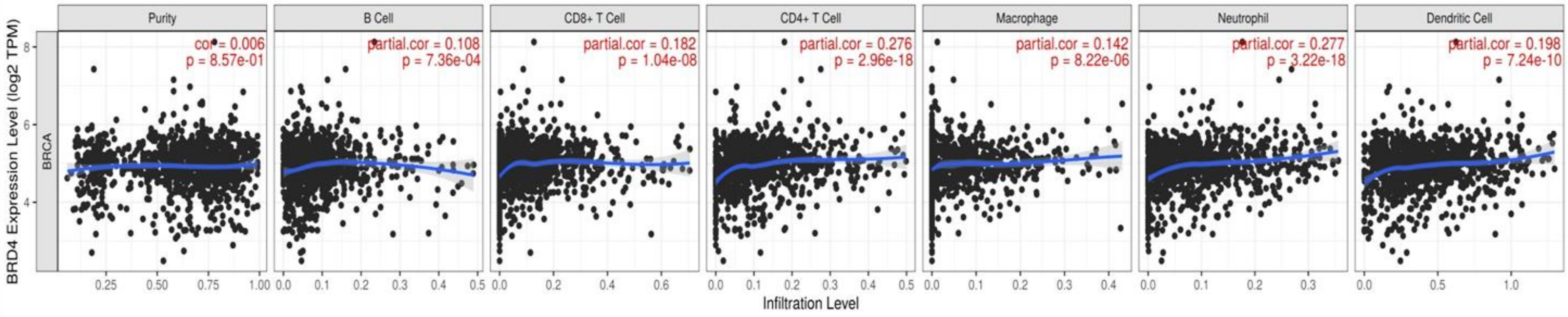

Supplementary figure S7

**RAC1**

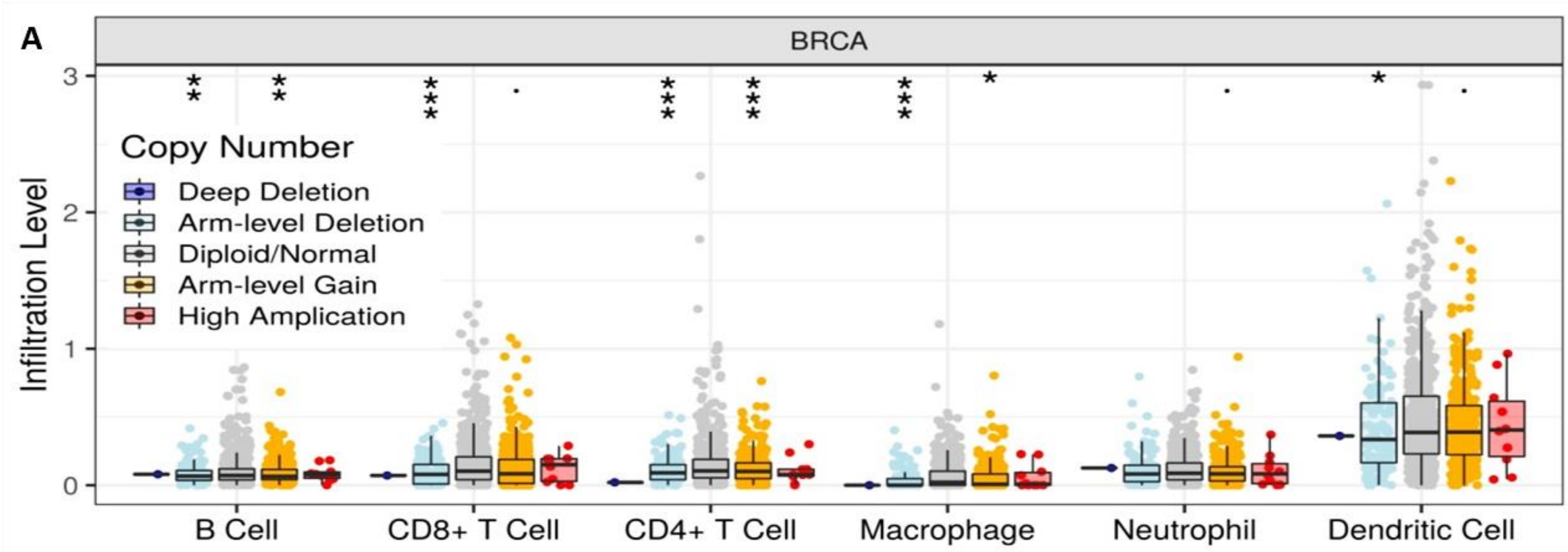


**BRD4**

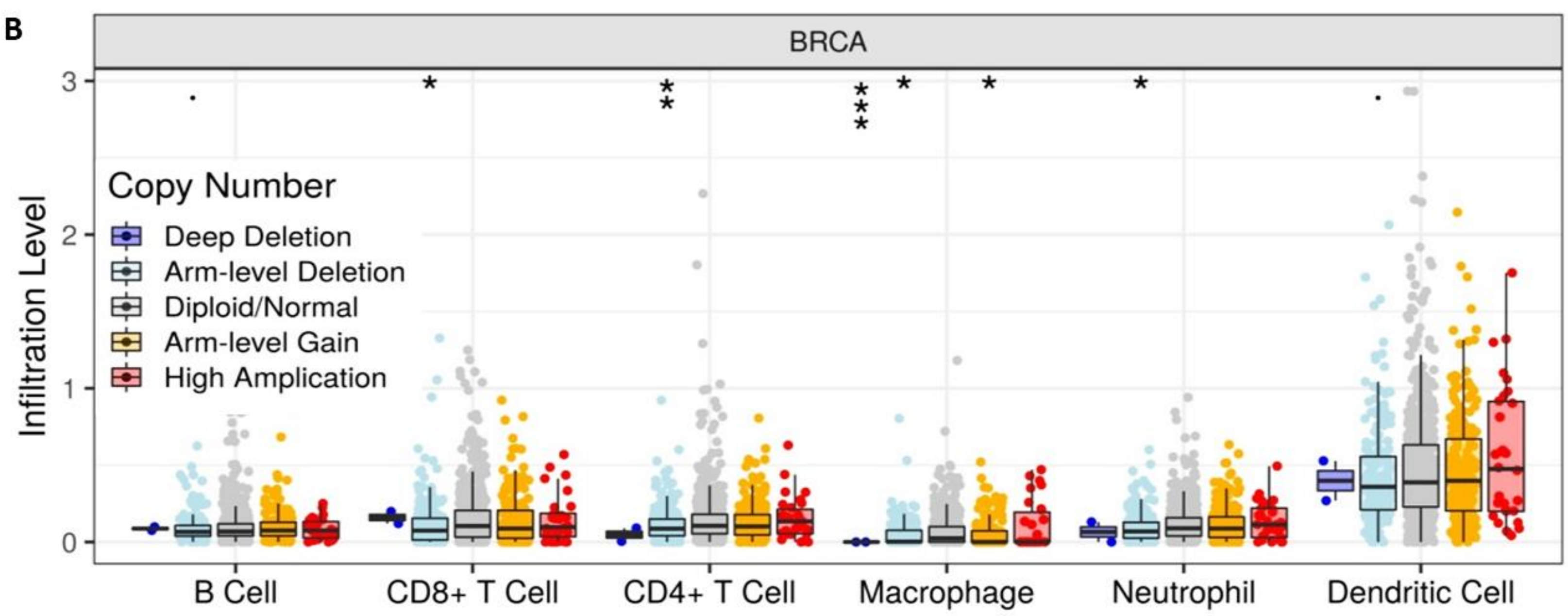

Supplement: Supplementary file 1 — Supplementary figures. [file ijbsv17p4474s1.pdf]
